# Development of an Autonomous Mobile Robot System Capable of Tracking Workers in a Factory Environment

**DOI:** 10.3390/s26196292

**Published:** 2026-10-04

**Authors:** Hyeongmuk Kang, Giseo Park, Sungmin Kim

**Affiliations:** School of Mechanical Engineering, University of Ulsan, Ulsan 44610, Republic of Korea; zollehxkt@mail.ulsan.ac.kr (H.K.); sungminkim@ulsan.ac.kr (S.K.)

**Keywords:** autonomous mobile robot, worker-following, ArUco marker, global path smoothing, dynamic window approach, factory environment

## Abstract

An autonomous mobile robot (AMR) that follows a worker can reduce the physical burden on the worker, but stable navigation remains difficult in the narrow and cluttered spaces of a factory, where the conventional dynamic window approach (DWA) tends to prefer high linear velocities and therefore turns with a large radius. This paper proposes a worker-following navigation system with three specific elements: a unique ArUco marker whose pose, obtained by solving the perspective-n-point problem, is transformed into the map frame to define the goal point; an A* search whose evaluation function includes the costmap cost, followed by waypoint pruning and cubic B-spline refinement of the resulting path; and an improved DWA that adds a corner-aware term regulating the linear velocity according to the heading change expected over the prediction horizon. The system is implemented on an AMR equipped with a 3-D light detection and ranging (LiDAR) and a camera, and is evaluated in a demonstration factory through two scenarios of 30 trials each. The proposed algorithm completes all 60 trials without a collision, whereas Nav2 and the fast marching method (FMM)-DWA record 22 and 17 collisions, respectively. The experimental results demonstrate that path-geometry-aware velocity control improves navigation stability and reduces collision risk in complex factory environments.

## 1. Introduction

In recent factory environments, the demand for autonomous mobile robots (AMRs) has increased to reduce workload and improve task efficiency. In particular, worker-following AMRs have the advantage of supporting tasks in real time at the location where the human worker needs assistance, such as logistics transportation and manufacturing [1,2].

For stable navigation of a worker-following AMR, various methods, such as the dynamic window approach (DWA), model predictive control (MPC) and the artificial potential field (APF) method, have been utilized [3]. Among them, DWA is suitable for the edge computing systems of AMRs because it requires relatively low computational cost while directly generating linear and angular velocity commands.

However, the tendency of the conventional DWA to prefer high linear velocities can increase the risk of collision in sharp turning sections or near obstacles [4]. Therefore, in factory environments where workers, equipment, and structures coexist, stable navigation requires adaptive linear velocity control according to the path condition and surrounding obstacles, rather than simply maintaining a high linear velocity.

### 1.1. Objectives

In this paper, an AMR is newly designed for factory environments with complex structures. It recognizes a specific ArUco marker and estimates, in real time, the position of the worker holding it, which is then used as the goal point of the navigation system.

**Figure 1:**
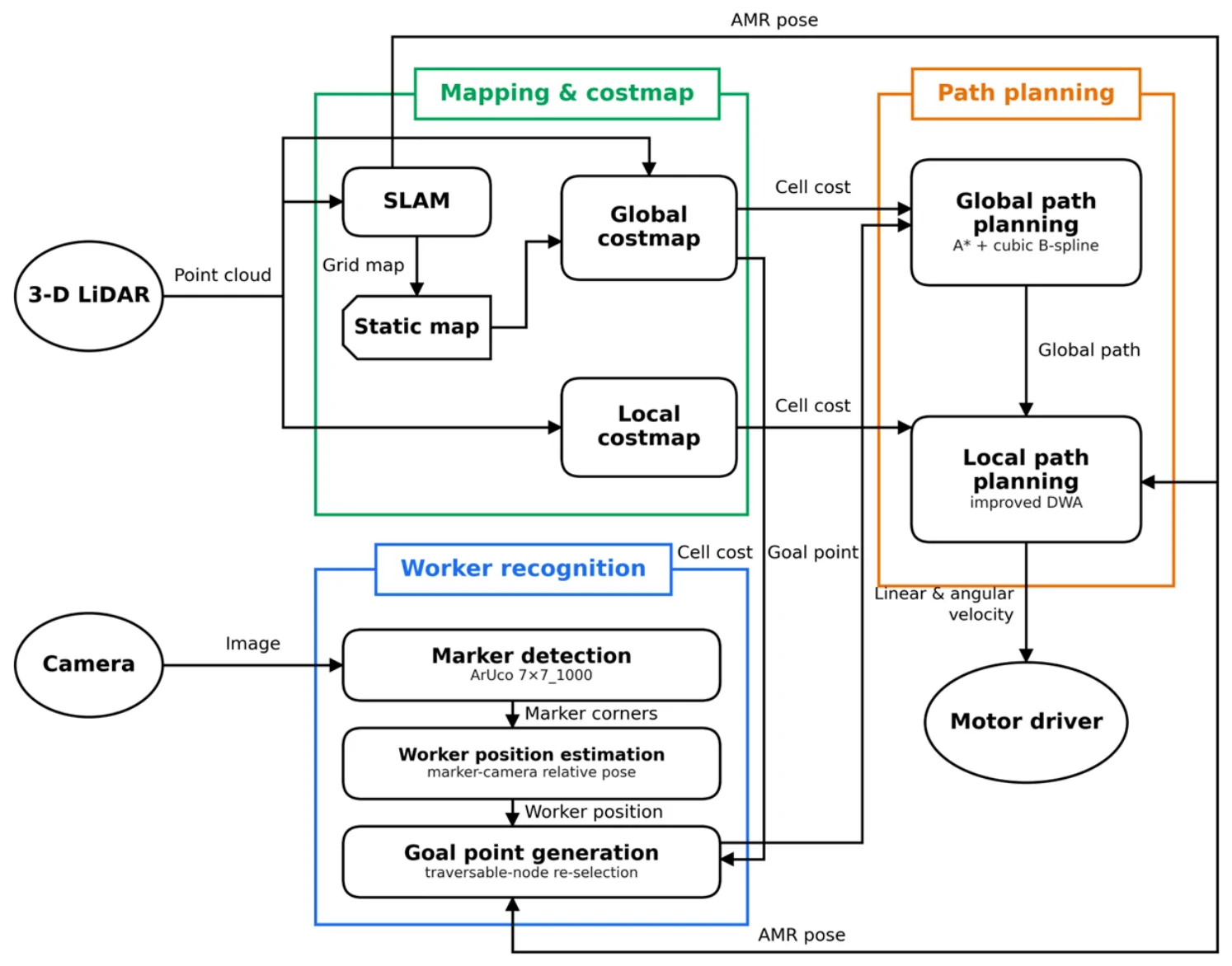
Data flow architecture of the proposed worker-following AMR.

**Figure 2:**
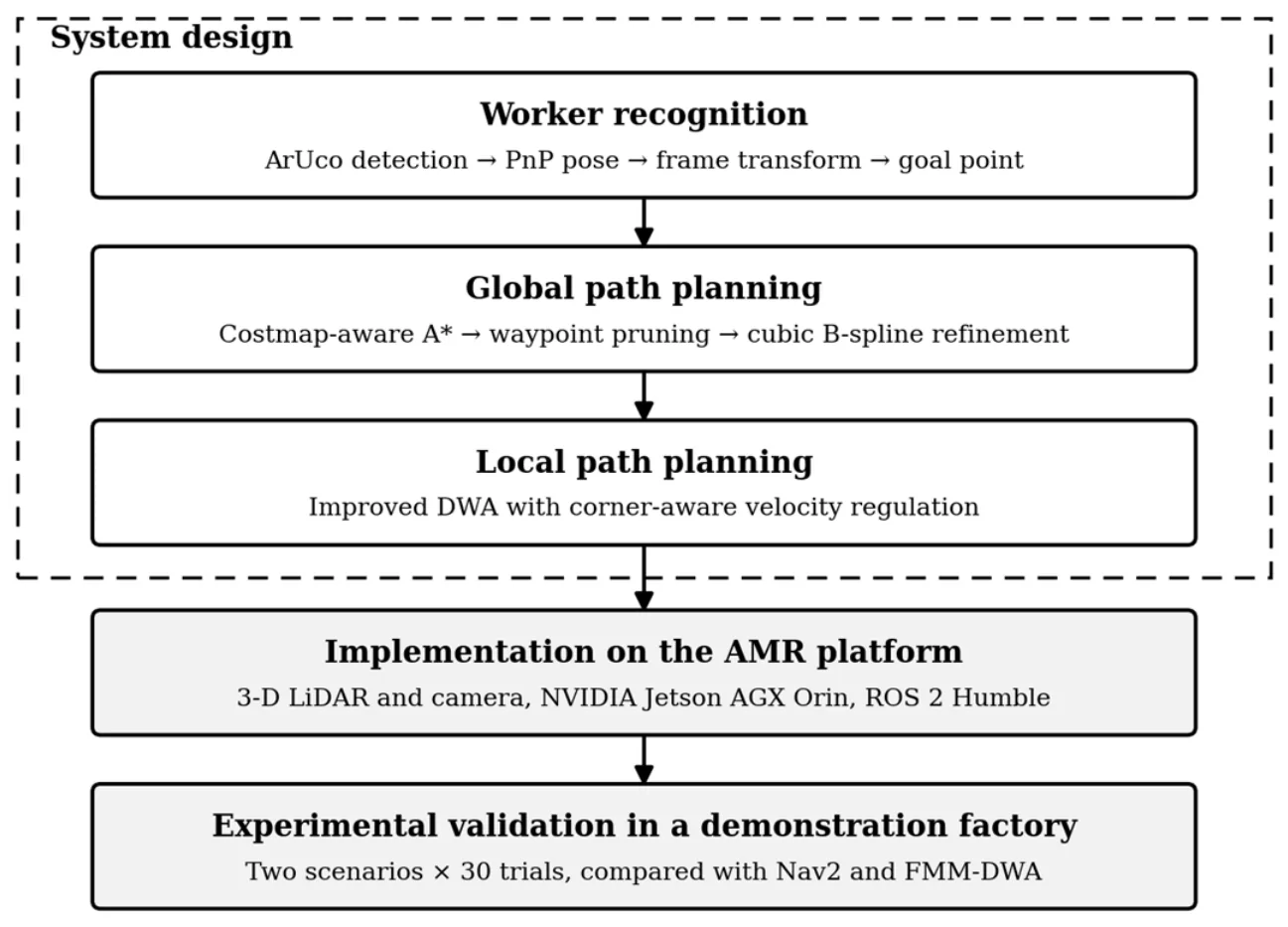
Overall research flow of this study.

A global path to that goal point is generated by an A* search on a costmap-based grid, and the resulting polyline is refined by waypoint pruning and cubic B-spline smoothing. The refined path is then tracked by an improved DWA for local path planning. In particular, the newly designed cost function of the DWA simultaneously considers the global-path-following performance, the ability to avoid surrounding obstacles, and the linear velocity variation required during turning. Finally, the generated velocity command is transmitted to the two main driving wheels. The overall structure of the proposed system is shown in Figure 1. Also, the overall flow of the research is summarized separately in Figure 2.

### 1.2. Contributions

The main contributions of this paper are as follows.

(1) Real-time worker recognition

A unique ArUco marker identifies the assigned worker, and the marker pose is transformed into the map frame so that the goal point is obtained without additional environmental infrastructure or a learned appearance model.

(2) Costmap-aware global path

The A* evaluation function is extended with the node cost of the global costmap, and the resulting polyline is refined by waypoint pruning and cubic B-spline smoothing, so that the reference path keeps a margin from the surrounding structures without unnecessary heading changes.

(3) Corner-aware local planning

A velocity regulation term based on the heading change expected over the prediction horizon is added to the DWA cost function, so that the AMR decelerates before curved or narrow sections while keeping a higher speed in straight sections.

The complete system is implemented on an AMR and evaluated in a demonstration factory against Nav2 and FMM-DWA.

## 2. Related Work

### 2.1. Global and Local Fusion Navigation

Liu and Zhang [5] proposed ASL-DWA, which generates a global path using an improved A* algorithm and adds the global path yaw angle to the DWA cost function. Wu et al. [6] proposed a hybrid algorithm combining smoothing A* and improved DWA for forklift automated guided vehicles (AGVs). They added the distance between the candidate trajectory and the global path to the DWA cost function so that the local path does not deviate significantly from the global path during obstacle avoidance. Bakker et al. [7] proposed a path-following controller that minimizes the distance and heading error relative to the desired path. However, this method mainly focuses on returning to the global path after obstacle avoidance and does not sufficiently consider stable path-following characteristics in narrow spaces or sections with sharp curvature changes.

### 2.2. Deep Learning-Based Navigation

Xue et al. [8] proposed a deep reinforcement learning-based mapless navigation method for mobile robots operating in volatile, uncertain, complex and ambiguous environments. The controller takes light detection and ranging (LiDAR) measurements together with the relative goal direction as inputs and directly outputs the linear and angular velocity commands, without requiring a prior map. Raj and Kos [9] proposed a reinforcement learning-based mobile robot controller using a deep Q-network (DQN). In this approach, the robot learns a control policy through trial and error while interacting with the environment. The cost function is designed to guide the robot toward a target point while avoiding obstacles, and the DQN selects the most appropriate action from the set of possible actions in the current state. Cao et al. [10] proposed a hybrid path-following controller that combines pure pursuit with deep reinforcement learning. Pure pursuit is responsible for steering control while the soft actor critic (SAC) algorithm learns an adaptive velocity control policy. Such methods offer advantages in improving adaptability to complex environmental changes and enabling more flexible decision-making compared to fixed rule-based control. However, most approaches heavily rely on simulation training, require large amounts of training data and careful reward function design, and have limitations in guaranteeing safety and robustness in real-world environments.

### 2.3. Direction Field-Based Navigation

Kapitanyuk et al. [11] proposed a guiding vector field-based controller for path-following control of nonholonomic mobile robots. It defines a direction field around the path and provides a reference direction at each position, enabling the robot’s heading to be controlled so that it follows the desired direction. Jin et al. [12] proposed a fast marching method (FMM)-DWA-based navigation method for autonomous following. In this approach, a direction field is defined over the map and incorporated into the trajectory evaluation of DWA, allowing candidate trajectories to be assessed based on how well their headings align with the reference direction. Such approaches improve navigation stability and allow the robot to adjust its heading in response to changes in the positions of surrounding obstacles. However, in complex environments or when obstacles change continuously, the field must be updated repeatedly, which increases the computational cost. The reviewed methods are summarized in Table 1.

## 3. Worker Recognition

In the proposed system, worker recognition provides the goal point required by the global path planner. In a factory environment, the AMR has to identify the specific worker that it is assigned to support, estimate the position of that worker on the navigation map, and update this position continuously while the worker keeps moving. Therefore, the recognition method has to satisfy three requirements simultaneously: a computational load low enough to be executed on an edge computer together with mapping and control, an output expressed in the same coordinate frame as the costmap, and the ability to recover the target after a temporary loss of observation.

Vision-based human detection using deep learning models provides robust detection performance, but an additional re-identification module is required to distinguish the assigned worker from other people appearing in the same scene [1]. Point cloud clustering methods based on LiDAR can track a human over a relatively long range, but the target is easily confused with equipment and structures in cluttered factory spaces [2]. Wearable radio devices avoid these problems, but they require additional infrastructure to be installed in the environment. Accordingly, a fiducial marker-based approach is employed in this paper [13,14]. Because each marker encodes a unique identifier, the identity of the target is obtained directly from the detection result without any appearance model or training dataset.

### 3.1. Marker-Based Worker Detection

**Figure 3:**
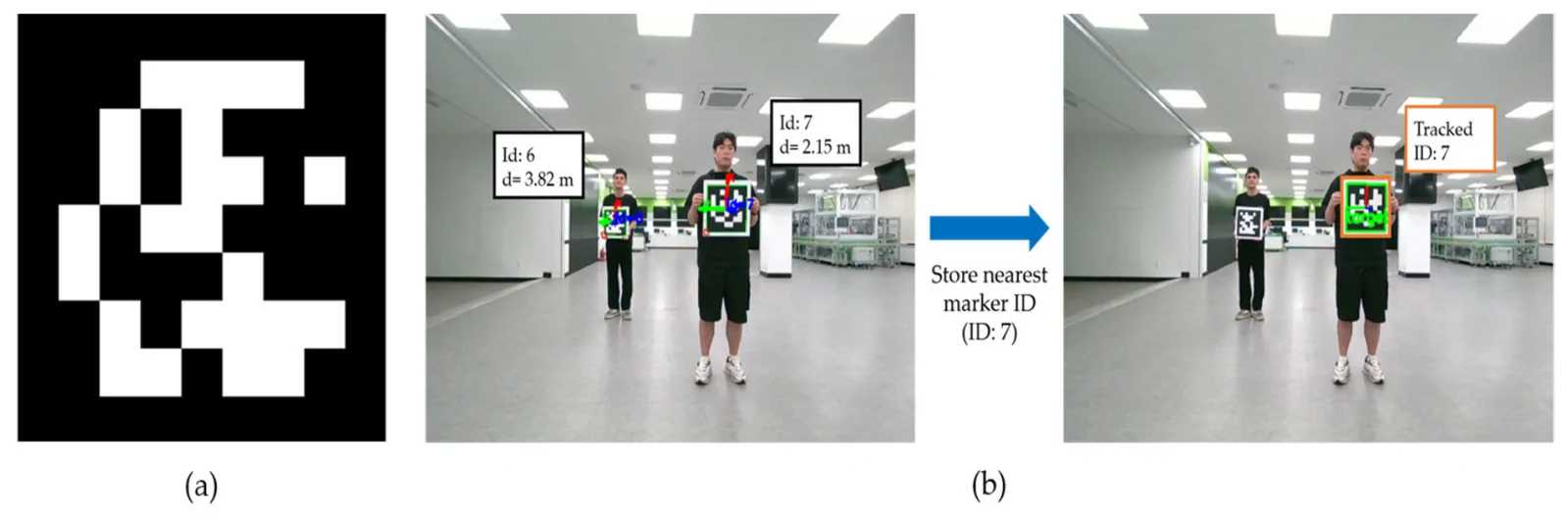
Worker recognition using ArUco markers: (**a**) ArUco marker display; (**b**) Detection of ArUco marker.

An ArUco marker consists of a black outer border and an inner binary matrix that encodes its identifier. As shown in Figure 3, an ArUco marker from the 7 × 7 (DICT_7X7_1000) dictionary is utilized in this paper, and it is held by the worker or attached to the back of the worker’s vest. Only the marker whose identifier is registered in advance is regarded as the target, so that other markers used in the same environment do not affect the navigation.

Accordingly, the marker detection success rate plays an important role in the worker-following AMR. To evaluate it, the detection rate is measured at various distances between the camera and the marker.

**Figure 4:**
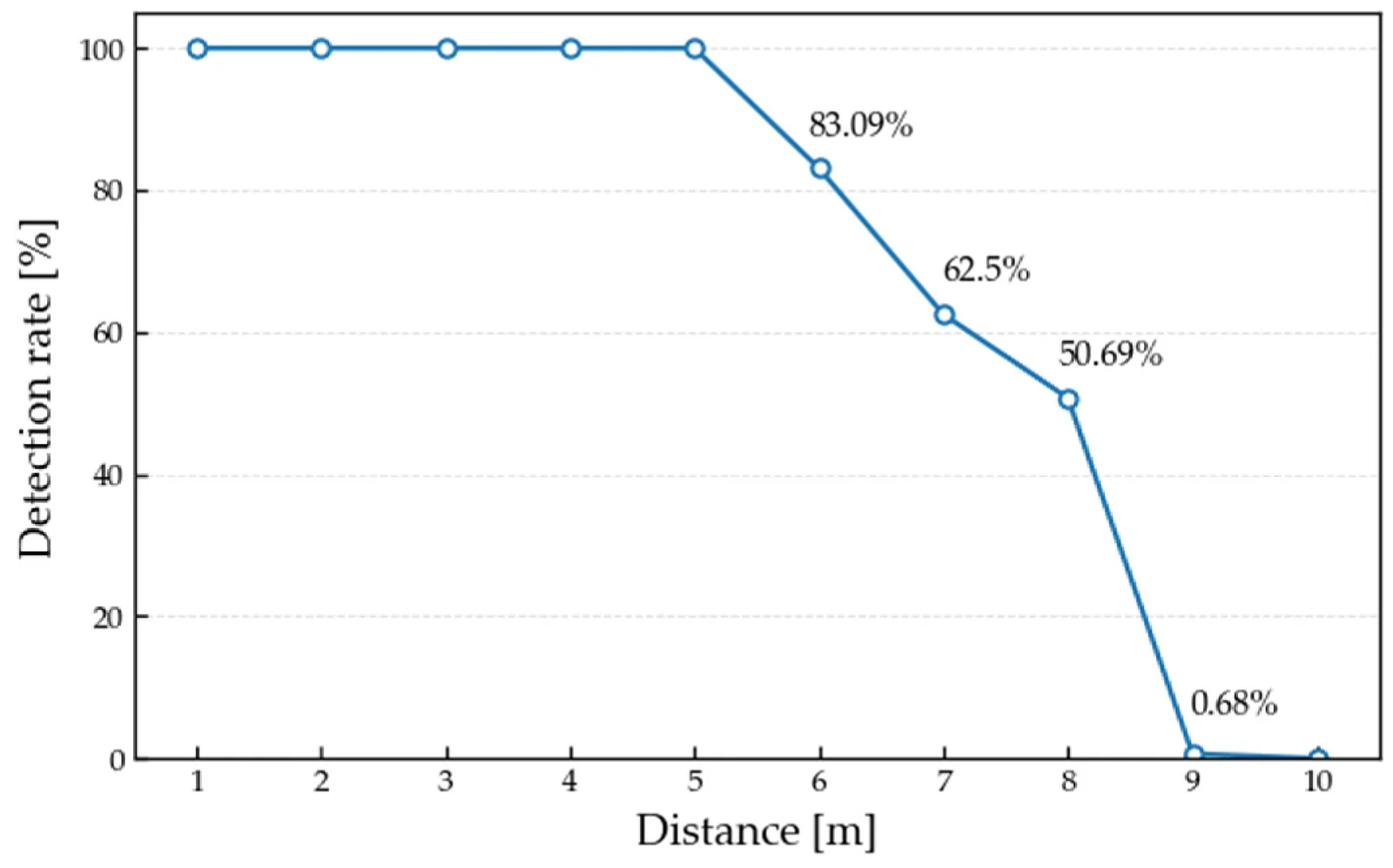
ArUco marker detection rates at different distances under high-illumination conditions.

As shown in Figure 4, the detection rate remains at 100% up to a distance of 5 m and then decreases rapidly, reaching 83.09% at 6 m, 62.5% at 7 m and 50.69% at 8 m, and falling below 1% beyond 9 m. These results were obtained indoors under the uniformly distributed and sufficiently bright illumination that characterizes the target environment. The horizontal illuminance at the marker plane, measured with a digital illuminance meter at ten positions distributed over the test area, is in the range 480–560 lx, which is consistent with the level recommended for general assembly work in industrial facilities.

Because the detection rate is affected by the lighting conditions as well as by the distance, a shorter effective range has to be expected under relatively low-light conditions. Nevertheless, a reliable detection range of about 5 m is sufficient for the worker-following task considered in this paper, in which the AMR is required to remain close to the worker.

### 3.2. Relative Position Estimation of the Worker

**Figure 5:**
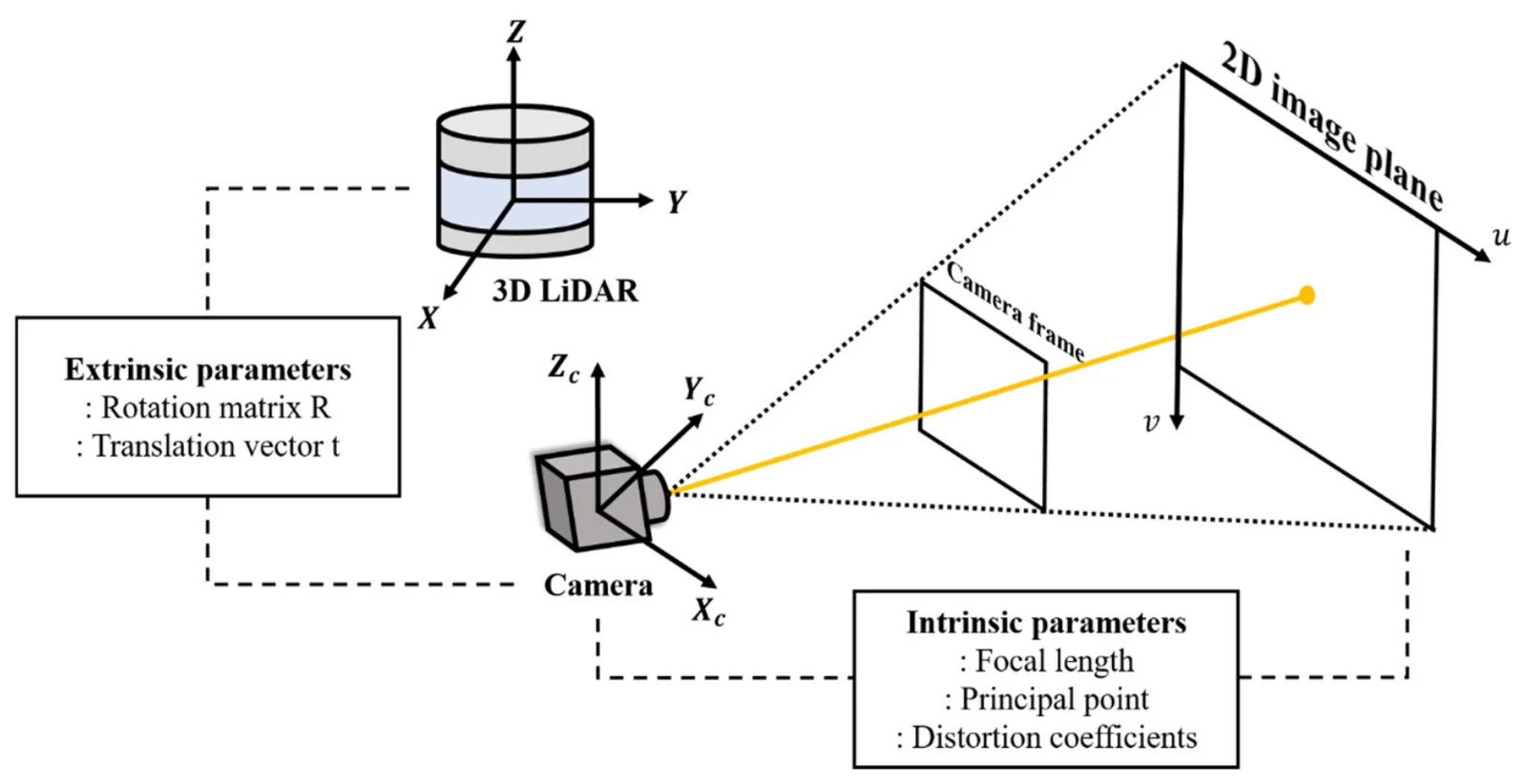
Camera and 3-D LiDAR calibration process.

As shown in Figure 5, the calibration process estimates the extrinsic parameters between the 3-D LiDAR and camera sensors by minimizing the error between the projected 3-D points in the LiDAR coordinate system and the 2-D reference points extracted from the image plane.

The relationship between a 3-D point in the LiDAR coordinate system and its corresponding 2-D pixel coordinate on the image plane can be expressed as follows.

(1) $$s\left[\begin{array}{c} u\\ v\\ 1 \end{array}\right]=\left[\begin{array}{ccc} f_{x} & 0 & c_{x}\\ 0 & f_{y} & c_{y}\\ 0 & 0 & 1 \end{array}\right]\left[\begin{array}{ccc} \begin{array}{c} r_{11}\\ r_{21}\\ r_{31} \end{array} & \begin{array}{c} r_{12}\\ r_{22}\\ r_{32} \end{array} & \begin{array}{cc} \begin{array}{c} r_{13}\\ r_{23}\\ r_{33} \end{array} & \begin{array}{c} t_{x}\\ t_{y}\\ t_{z} \end{array} \end{array} \end{array}\right]\left[\begin{array}{c} X_{L}\\ Y_{L}\\ \begin{array}{c} Z_{L}\\ 1 \end{array} \end{array}\right]$$

Here, (*X_L_*, *Y_L_*, *Z_L_*) represents a 3-D point in the LiDAR coordinate system, and (*u*, *v*) denotes the pixel coordinates on the image plane. In addition, *f_x_*_,*y*_ represent the focal lengths of the camera, while *c_x_*_,*y*_ denote the principal point coordinates. In addition, *r_ij_* is each element of the rotation matrix, and *t_x_*_,*y*,*z*_ are the components of the translation vector. The variable *s* is the scale factor generated when a 3-D point is projected onto the image plane.

The intrinsic parameters, namely the focal lengths and the principal point coordinates, depend only on the camera itself and are obtained beforehand by a checkerboard calibration. The extrinsic parameters, namely the rotation matrix and the translation vector, describe the rigid-body transformation from the LiDAR coordinate system to the camera coordinate system. The rotation matrix encodes the relative orientation of the two sensors, and the translation vector expresses the position of the origin of the LiDAR frame in the camera frame.

In the actual calibration process, the projected coordinates may not perfectly coincide with the reference points extracted from the image because of sensor measurement errors and reference point detection errors. Therefore, the extrinsic parameters *R*,*t* are estimated so that the error over all corresponding points is minimized. This can be formulated as the following least-squares problem.

(2) $$\underset{R,t}{\min}\sum_{i=1}^{N}{\left\|\left[\begin{array}{c} u_{i}\\ v_{i}\\ 1 \end{array}\right]-\frac{1}{s_{i}}\left[\begin{array}{ccc} f_{x} & 0 & c_{x}\\ 0 & f_{y} & c_{y}\\ 0 & 0 & 1 \end{array}\right]\left[\begin{array}{ccc} \begin{array}{c} r_{11}\\ r_{21}\\ r_{31} \end{array} & \begin{array}{c} r_{12}\\ r_{22}\\ r_{32} \end{array} & \begin{array}{cc} \begin{array}{c} r_{13}\\ r_{23}\\ r_{33} \end{array} & \begin{array}{c} t_{x}\\ t_{y}\\ t_{z} \end{array} \end{array} \end{array}\right]\left[\begin{array}{c} X_{L,i}\\ Y_{L,i}\\ \begin{array}{c} Z_{L,i}\\ 1 \end{array} \end{array}\right]\right\|}^{2}$$

Here, *N* denotes the number of corresponding points used for calibration. Once the extrinsic parameters have been obtained, the pose of the detected ArUco marker with respect to the camera frame is estimated by solving the perspective-n-point (PnP) problem using the four detected marker corners and the known marker size. The resulting translation vector, which corresponds to the position of the marker center in the camera frame, is regarded as the position of the worker *p_c_*.

### 3.3. Goal Point Generation

The estimated worker position has to be converted into the map frame. The transformation from the base frame to the map frame is obtained from the AMR pose estimated by simultaneous localization and mapping (SLAM), whereas the transformation from the camera frame to the base frame is fixed by the sensor mounting geometry. Hence, the worker position in the map frame can be expressed as follows.

Four coordinate frames are involved in this process. The marker pose is first obtained in the camera frame from the PnP solution described above, then mapped into the base frame by the constant transformation fixed by the sensor mounting geometry, and finally into the map frame by the transformation provided by the SLAM localization, which changes as the AMR moves. The complete chain is therefore map frame $T_{\mathrm{base}}^{\mathrm{map}}\left(x_{r},\;y_{r},\;\theta_{r}\right)\to$ base frame $T_{\mathrm{cam}}^{\mathrm{base}}\to$ camera frame *p_c_*→ marker.

(3) $$P_{w}=T_{\mathrm{base}}^{\mathrm{map}}\left(x_{r},\;y_{r},\;\theta_{r}\right)T_{\mathrm{cam}}^{\mathrm{base}}p_{c}$$

Here, (*x_r_*, *y_r_*, *θ_r_*) denote the pose of the AMR in the map frame. However, the worker holding the marker is simultaneously detected as an obstacle by the 3-D LiDAR. As a result, the goal point derived from the marker can be located inside the obstacle inflation region of the global costmap described in Section 3.1, and in this case a path to the goal point cannot be generated. To prevent this, when the cost of the goal node *C_g_*(*n*) exceeds the threshold of the obstacle inflation region *C_g_*_,th_, the nearest traversable node around the marker position is re-selected as the goal point as follows.

(4) $$P_{\mathrm{goal}}=\arg\underset{p_{n}\;\in \;N_{\mathrm{free}}}{\min}\left\|p_{n}-P_{w}\right\|$$

Here, *p_n_* denotes the position of node *n* in the global costmap, and *N*_free_ = {*n*| *C_g_*(*n*) < *C_g_*_,th_} is the set of traversable nodes, that is, the nodes whose global costmap cost is below the threshold of the obstacle inflation region. Through this procedure, the goal point is always placed in a traversable region located as close as possible to the worker.

The goal point is updated whenever the marker is detected, so that the global path is regenerated according to the movement of the worker. When the marker temporarily leaves the field of view of the camera, the most recently estimated goal point is retained and the AMR continues to follow the existing global path. Once the marker re-enters the field of view, the target is immediately recovered by its unique identifier.

## 4. Global Path Planning Strategy

### 4.1. Global Costmap Generation

A SLAM algorithm is used to generate the factory environment map while estimating the position and orientation of the AMR in real time [15,16]. The arrangement of the equipment is reconfigured between production campaigns, so the SLAM map has to be built on site and rebuilt whenever the layout changes.

Based on the SLAM Toolbox package for robot operating system 2 (ROS 2), a 2-D occupancy grid map of the demonstration factory environment is generated. An occupancy grid map divides the surrounding environment into nodes of equal size and stores the occupancy state of each node. Accordingly, free, occupied and unknown regions can be classified based on the 2-D scan data acquired from the LiDAR.

A costmap can generate an obstacle-avoidance region by accounting for the robot footprint and a safety margin around detected obstacles, thereby reducing collision risk. For this purpose, a distance field is constructed on the 2-D map. Previous studies have employed methods such as the FMM, Euclidean signed distance fields, and Dijkstra-based methods to calculate and store obstacle-distance information for each node.

The global costmap incorporates obstacle position information into all nodes of the 2-D map generated through SLAM. The detailed process is presented in Algorithm 1. After removing point cloud data corresponding to the floor and ceiling, the nodes corresponding to the remaining obstacle points are defined as source nodes. Then, the distance information is propagated through adjacent nodes using a Dijkstra-based method, which determines the shortest path by accumulating the transition costs between adjacent nodes.

**Figure 6:**
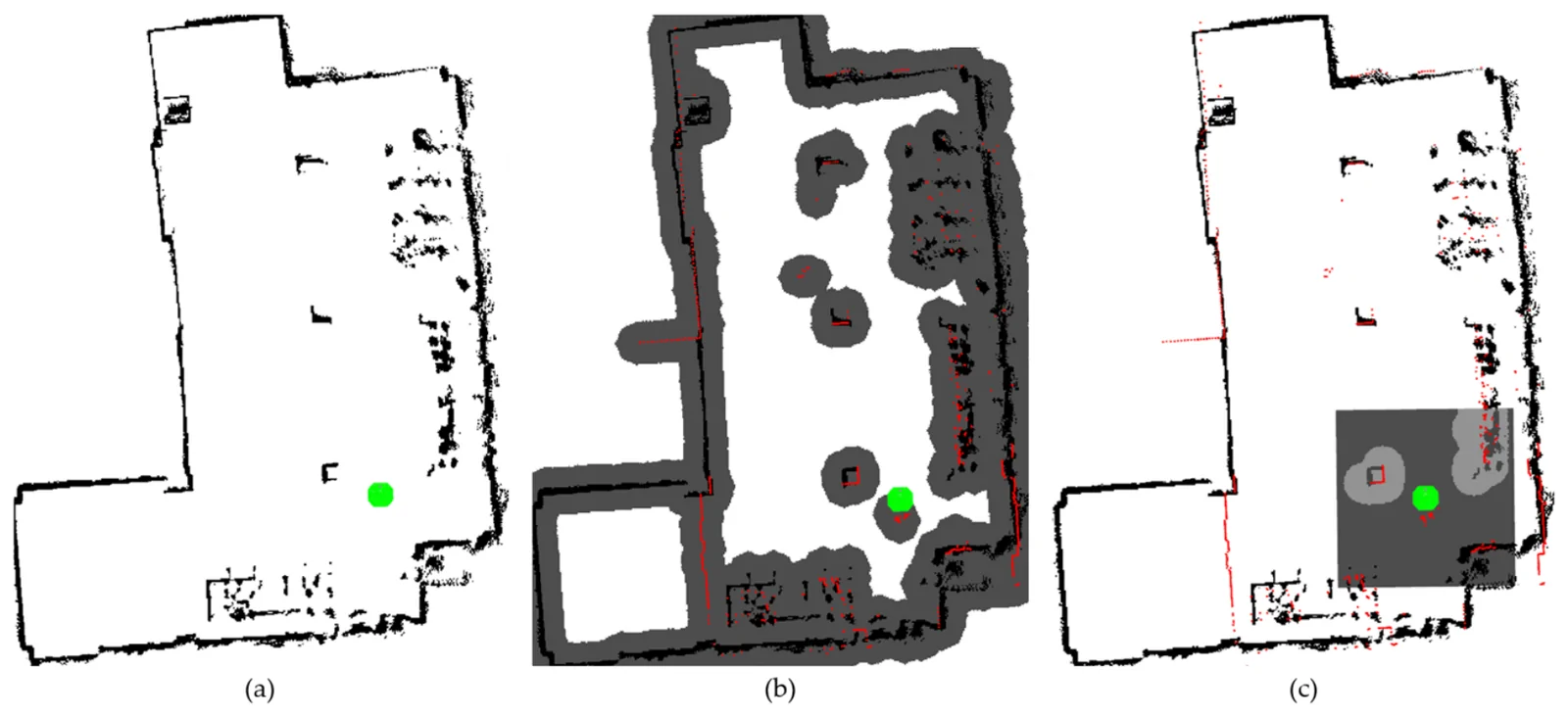
Demonstration factory environment: (**a**) Original map; (**b**) Global costmap; (**c**) Local costmap.

Based on the generated distance field, an additional cost is assigned to nodes whose distance from the nearest obstacle is less than or equal to *d_max_*. In contrast, free nodes located beyond *d_max_* are assigned a cost of zero and are regarded as traversable regions. The resulting global costmap over the entire map is shown in Figure 6.

The computational cost of Algorithm 1 is dominated by the Dijkstra-based propagation of lines 5–9. For a grid with |*V*| nodes and |*E*| edges, the propagation with a binary heap runs in O(|*E*|*log*|*V*|); because the grid is eight-connected, |*E*| ≤ 8|*V*|, so the bound reduces to O(|*V*|*log*|*V*|). The initialization of lines 3–4 and the assignment of lines 10–11 are both O(|*V*|), and the memory requirement is O(|*V*|). The global costmap is therefore generated in O(|*V*|*log*|*V*|) time, and this cost is incurred once per global costmap update period rather than at every control cycle.
**Algorithm 1.** Global costmap generation**Input:** point cloud *P*, occupancy grid map *M*, inflation distance *d_max_*, constant cost *C_g_***Output:** global costmap *C*1:    remove from *P* the points belonging to the floor and to the ceiling2:    *S* ← { *n* ∈ *M* | *n* contains at least one remaining point of *P* }3:    **for all** *n* ∈ *M* **do** *D*(*n*) ← ∞4:    **for all** *n* ∈ *S* **do** *D*(*n*) ← 0; insert *n* into the priority queue *Q*5:    **while** *Q* is not empty **do**6:           *n* ← extract-min(*Q*)7:           **for all** neighbors *n*′ of *n* **do**8:                   **if** *D*(*n*) + *c*(*n*, *n*′) < *D*(*n*′) **then**9:                          *D*(*n*′) ← *D*(*n*) + *c*(*n*, *n*′); update *n*′ in *Q*10:  **for all** *n* ∈ *M* **do**11:           **if** *D*(*n*) ≤ *d_max_* **then** *C*(*n*) ← *C_g_* **else** *C*(*n*) ← 012:  **return** *C*

### 4.2. A* Algorithm

The A* algorithm is a representative graph-based path planning algorithm that searches for the path with the lowest cost from a start point to a goal point. It combines the optimal path search capability of Dijkstra’s algorithm with the direction search efficiency of greedy best-first search. The evaluation function of the conventional A* algorithm is defined as

*f*(*n*) = *g*(*n*) + *h*(*n*). (5)

Here, *f*(*n*), *g*(*n*) and *h*(*n*) represent the total evaluation cost of the current node *n*, the accumulated cost from the start node to the current node and the heuristic cost from the current node to the goal node, respectively.

To generate a minimum cost path to the goal, the node with the smallest *f*(*n*) value is selected and preferentially expanded. The accumulated path cost *g*(*n*) is calculated as the sum of the movement costs incurred from the start node to the current node. In this paper, movement costs are assigned for eight directions based on the relative distance between adjacent grid cells. This cost assignment enables an eight-directional search while accurately reflecting the movement distance in a grid-based environment. To obtain a relatively accurate and efficient heuristic estimate, a Euclidean distance-based function that supports movement in eight directions is employed.

In this paper, to consider both AMR radius and the minimum range of the LiDAR, the extended evaluation function is designed as follows.

*f*(*n*) = *g*(*n*) + *h*(*n*) + *C_g_*(*n*) (6)

**Figure 7:**
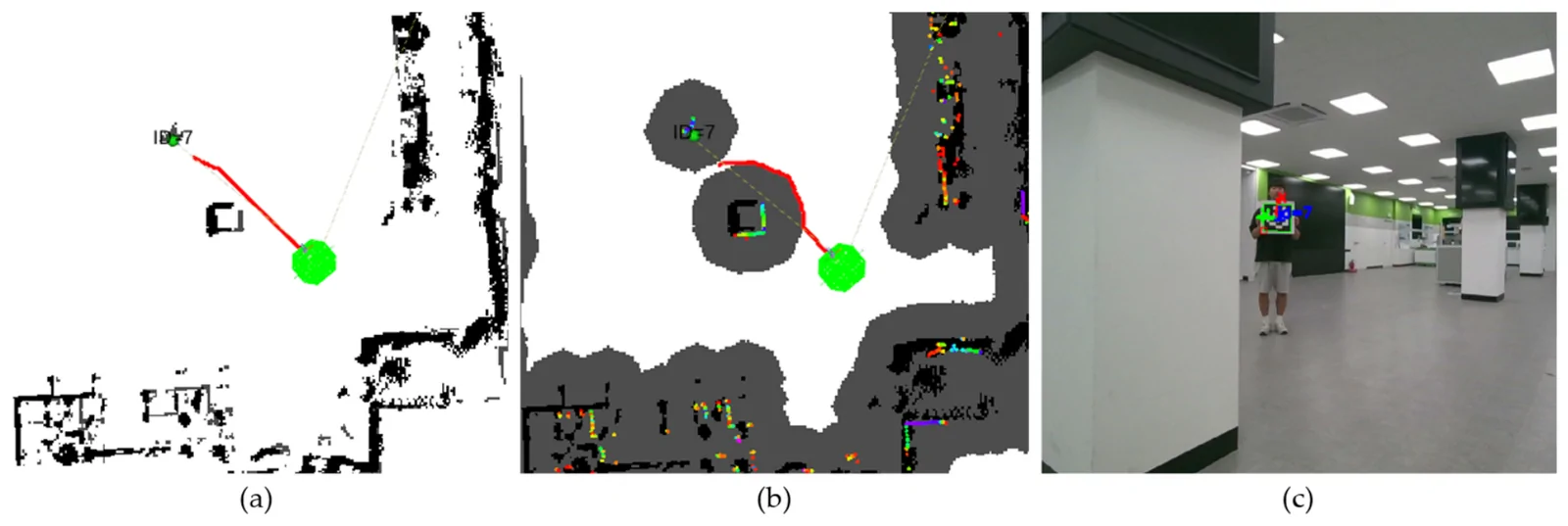
Global path results: (**a**) Before and (**b**) After applying the obstacle avoidance region; (**c**) Actual factory environment with ArUco marker.

Here, *C_g_*(*n*) denotes the cost of a node in the costmap. A constant cost value is assigned to all nodes located within the obstacle inflation region, whereas nodes outside this region are assigned a cost of zero. The effect of this additional term is illustrated in Figure 7. Without it, the global path passes close to the surrounding structures, as shown in Figure 7a, whereas with it the path is kept away from them, as shown in Figure 7b. Figure 7c shows the corresponding scene in the demonstration factory, in which the worker holds the registered ArUco marker. The path obtained in this way still consists of a sequence of line segments connecting neighboring nodes.

### 4.3. Curved Path Generation

The generated path may include redundant intermediate waypoints, particularly in sections where several consecutive waypoints are positioned along nearly the same direction. To address this issue, the piecewise linear path has to be transformed into a smooth curved path.

Among various path smoothing methods, such as B-spline smoothing [17], Bézier curves [18] and Theta* [19], B-spline smoothing is considered suitable for refining the costmap-based global path and is employed in the proposed algorithm.

First, a pruning procedure is performed to eliminate waypoints that have little influence on the overall path geometry. For three consecutive waypoints *a*, *b* and *c*, the collinearity of the corresponding vectors $\vec{\mathrm{ab}}$ and $\vec{\mathrm{bc}}$ is evaluated using their 2-D cross product. When the magnitude of the cross product is smaller than a predefined threshold *ε*, the three waypoints are considered to be approximately aligned. This condition is expressed as follows.

(7) $$\left|\vec{\mathrm{ab}}\times \vec{\mathrm{bc}}\right|<\varepsilon$$

If this condition is satisfied, waypoint *b* is removed. Repeating this procedure along the entire path reduces the number of unnecessary waypoints while preserving the principal navigation direction.

A cubic B-spline constructs a continuous curve from a local set of control points and is therefore suitable for reducing abrupt directional changes in the A*-based path. The general form of the B-spline curve is given by

(8) $$Q\left(t\right)=\sum_{i=0}^{3}P_{i}N_{i,r}(t)$$

where *P_i_* is the *i*-th control point and *N_i_*_,*r*_(*t*) denotes the corresponding B-spline basis function of degree *r*, with *r* = 3 for a cubic B-spline. For a cubic B-spline, each curve segment is determined by four neighboring control points. The segment can be represented in matrix form with *t* ∈ [0, 1] as

(9) $$Q\left(t\right)=\frac{1}{6}\left[\begin{array}{cc} \begin{array}{cc} t^{3} & t^{2} \end{array} & \begin{array}{cc} t & 1 \end{array} \end{array}\right]\left[\begin{array}{cccc} -1 & 3 & -3 & 1\\ 3 & -6 & 3 & 0\\ -3 & 0 & 3 & 0\\ 1 & 4 & 1 & 0 \end{array}\right]\left[\begin{array}{c} P_{0}\\ P_{1}\\ P_{2}\\ P_{3} \end{array}\right].$$

The generated cubic B-spline segments are connected in sequence. Next, let *M* denote the total number of control points. The resulting global path can be mathematically expressed as

(10) $$P_{\mathrm{path}}=\overset{M-4}{\underset{k=0}{⋃}}\left\{Q_{k}\left(t\right)|t\in \left[0,\;1\right]\right\}$$

where *Q_k_*(*t*) represents the cubic B-spline segment constructed from the *k*-th group of four neighboring control points. Through this two-stage refinement process, the node-based polyline generated by the A* algorithm can be transformed into a continuous curved path without unnecessary directional changes [20]. For use by the local planner, the continuous curve *P_path_* is uniformly resampled at a fixed arc-length interval, which yields the discrete waypoint sequence {(*x_p_*_,*i*_, *y_p_*_,*i*_)}, *i* = 1,2,⋯,*M_p_*. Here *M_p_* denotes the number of resampled waypoints.

## 5. Local Path Planning Strategy

### 5.1. Local Costmap Generation

The local costmap assigns and publishes cost values only to the nodes within a user-defined region of interest (ROI) centered on the AMR, to reduce the processing burden and enable the controller to respond rapidly. An example of the resulting local costmap is shown in Figure 6c. Here, the update period of the global costmap is set to 0.5 s, whereas that of the local costmap is set to 0.15 s.

The local costmap is generated by the same procedure as the global costmap, with three differences: the grid is centred on the current AMR position (*x_r_*, *y_r_*) and is restricted to the ROI; the distance field is obtained by a Euclidean distance transform over the ROI rather than by a graph search over the entire map; and the layer is republished at the shorter update period given above, so that newly observed obstacles are reflected without rebuilding the global layer.

### 5.2. Improved DWA

**Figure 8:**
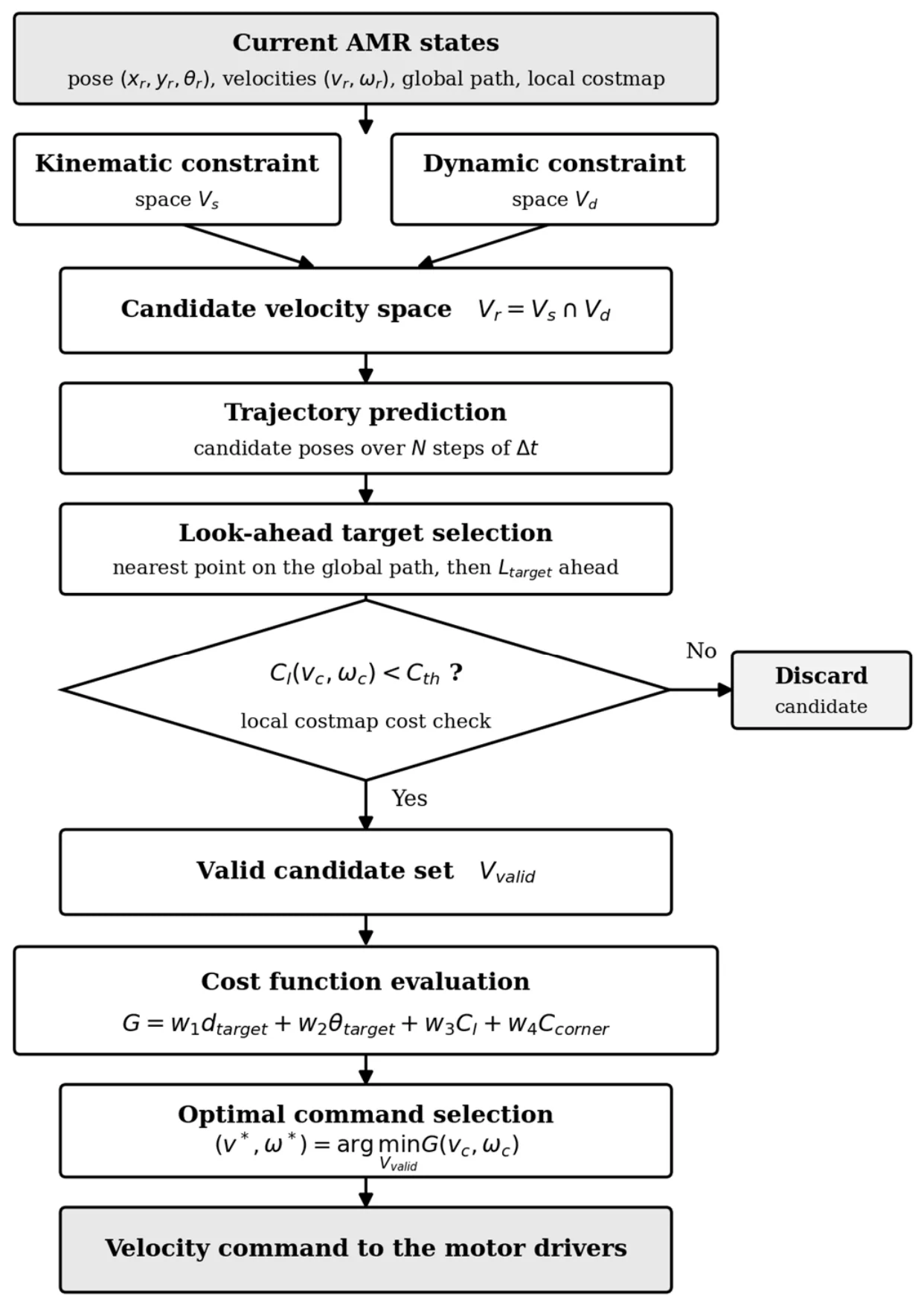
Flowchart of the proposed improved DWA.

The DWA is a local planning method that generates feasible linear and angular velocity candidates over a specified time horizon by considering the current velocity and dynamic constraints of the AMR. As shown in Figure 8, both the optimal linear and angular velocity commands can be determined based on the current AMR states.

The conventional DWA generally considers the distance to the target point, the heading error relative to the target direction and the preference for a higher linear velocity. Under these criteria, the AMR may travel toward the target at a high speed, thereby increasing the risk of collision with nearby obstacles.

Figure 8 illustrates the overall flow of the DWA proposed in this paper. The cost function consists of four evaluation terms, which enable the AMR to safely navigate to the target point.

#### 5.2.1. Candidate Velocity Set

The kinematic constraint space *V_s_* defines the overall allowable range of linear and angular velocities that the AMR can generate. In this paper, these velocity limits are predefined according to the operating conditions of the AMR. This prevents excessively high velocity commands from being generated and reduces the risk of collision. Accordingly, *V_s_* can be defined as the set of velocity pairs that satisfy the specified limits.

*V_s_* = {(*v_c_*, *ω_c_*)| *v_min_* ≤ *v_c_* ≤ *v_max_ and ω_min_* ≤ *ω_c_* ≤ *ω_max_*} (11)

Here, *v_min_*_,*max*_ and *ω_min_*_,*max*_ are the minimum and maximum linear and angular velocities, respectively.

In addition, the dynamic constraint space *V_d_* defines the range of linear and angular velocities that the AMR can reach within a single control interval ∆*t* based on its current motion states:

*V_d_* = {(*v_c_*, *ω_c_*)| *v_r_* − *a_v_*∆*t* ≤ *v_c_* ≤ *v_r_* + *a_v_*∆*t and ω_r_* − *a_ω_*∆*t* ≤ *ω_c_* ≤ *ω_r_* + *a_ω_*∆*t*} (12)

where the current linear and angular velocities of the AMR are denoted by *v_r_* and *ω_r_*, respectively, while *a_v_* and *a_ω_* represent the maximum linear and angular accelerations, respectively.

The candidate velocity space *V_r_* is defined as the intersection of the kinematically feasible velocity space *V_s_* and the dynamically reachable velocity space *V_d_*:

*V_r_* = {(*v_c_*, *ω_c_*)| (*v_c_*, *ω_c_*) ∈ (*V_s_* ∩ *V_d_*)}. (13)

Therefore, only velocity pairs that satisfy both the overall velocity limits of the robot and the velocities dynamically reachable from its current state are included in *V_r_*.

Based on the current pose, the future poses of the AMR can be estimated using the candidate linear and angular velocities generated from *V_r_*. When candidate linear and angular velocities are maintained over a specified period ∆*t*, the resulting robot pose can be calculated as follows.

*x_c_*_,*k*+1_ = *x_c_*_,*k*_ + *v_c_*∆*tcosθ_c_*_,*k*_ (14)

*y_c_*_,*k*+1_ = *y_c_*_,*k*_ + *v_c_*∆*tsinθ_c_*_,*k*_ (15)

*θ_c_*_,*k*+1_ = *θ_c_*_,*k*_ + *ω_c_*∆*t* (16)

Here, *x_c_*_,*k*_, *y_c_*_,*k*_ and *θ_c_*_,*k*_ denote the candidate position and orientation of the AMR at the *k*-th time step, respectively.

**Figure 9:**
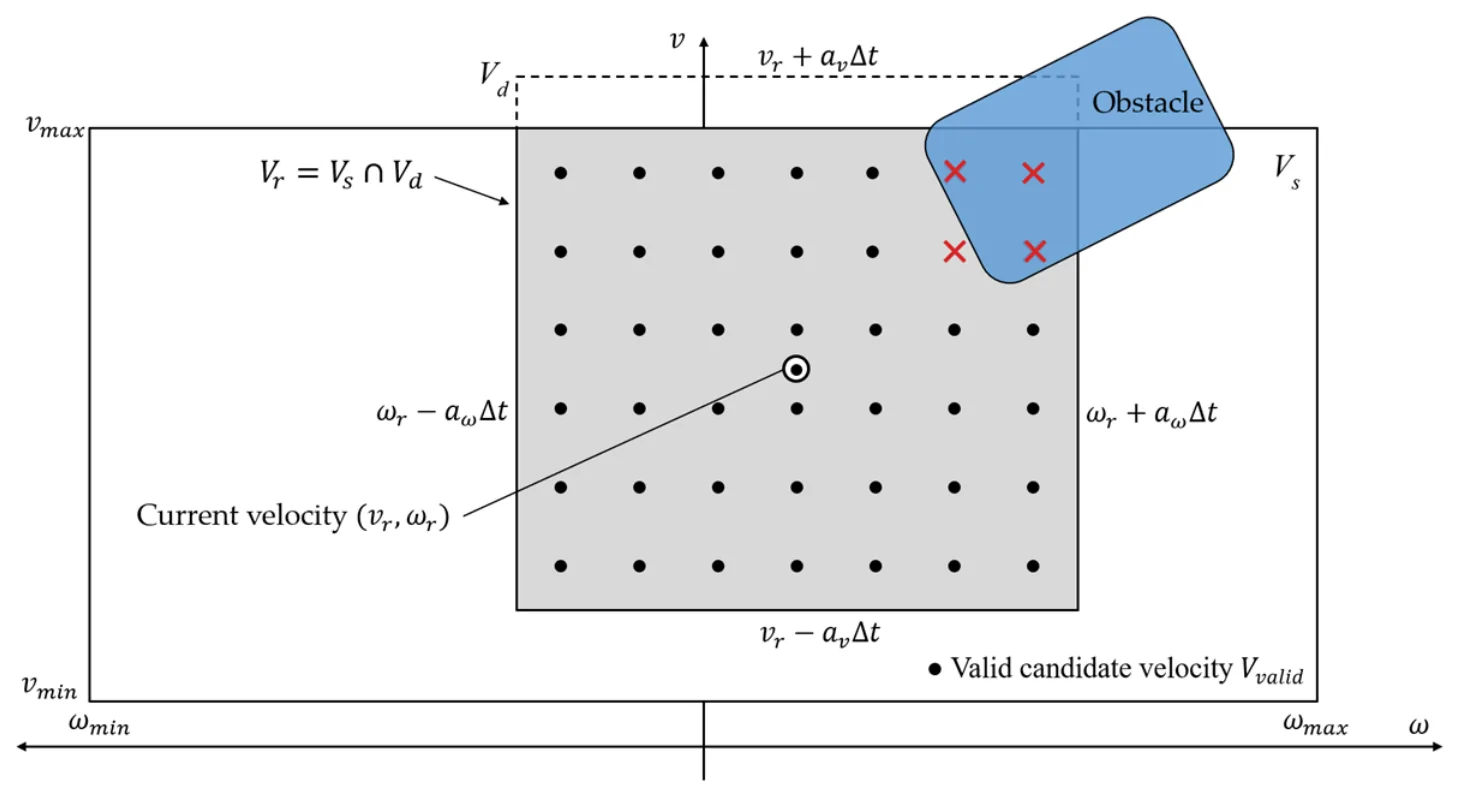
Visualization of candidate velocity set.

In this paper, collision risk is determined using obstacle-occupied nodes in the costmap, as shown in Figure 9. If any estimated pose enters a node containing an obstacle, the corresponding trajectory is regarded as having a collision risk. Its linear and angular velocity pairs are removed through hard filtering [21,22].

#### 5.2.2. Look-Ahead Target Selection

The index of the point on the global path that is closest to the current AMR position is obtained as follows.

(17) $$i_{\mathrm{near}}=\arg\;\underset{i}{\min}\sqrt{{\left(x_{p,i}-x_{r}\right)}^{2}+{\left(y_{p,i}-y_{r}\right)}^{2}}$$

Here, the corresponding point on the global path is denoted by *P_near_* = (*x_near_*, *y_near_*). Then, the look-ahead target is selected by accumulating the distances between consecutive coordinate points along the global path, starting from the corresponding point.

(18) $$i_{\mathrm{target}}=\min\left\{i|i\geq i_{\mathrm{near}}\;\mathrm{and}\;\sum_{j=i_{\mathrm{near}}}^{i-1}\sqrt{{\left(x_{p,j}-x_{p,j+1}\right)}^{2}+{\left(y_{p,j}-y_{p,j+1}\right)}^{2}}\geq L_{\mathrm{target}}\right\}$$

The selected index *i_target_* is then converted into the corresponding coordinate data, yielding the following result: *P_target_* = (*x_target_*, *y_target_*). The AMR moves toward the look-ahead target selected on the global path. When the distance between the AMR and the marker becomes less than a certain threshold, no additional look-ahead target can be generated, and the AMR consequently stops.

#### 5.2.3. Cost Function on DWA

The DWA algorithm considers the determined look-ahead target and candidate poses and ultimately outputs the linear and angular velocity commands to be applied to the AMR [23,24,25]. The cost function proposed in this paper is defined as follows.

*G*(*v_c_*, *ω_c_*) = *w*_1_*d_target_*(*v_c_*, *ω_c_*) + *w*_2_*θ_target_*(*v_c_*, *ω_c_*) + *w*_3_*C_l_*(*v_c_*, *ω_c_*) + *w*_4_*C_corner_*(*v_c_*, *ω_c_*) (19)

Here, *w* denotes a user-defined weight of each cost term. The first cost function term *d_target_* represents the distance between the look-ahead target and the estimated candidate pose:

(20) $$d_{\mathrm{target}}=\sqrt{{\left(x_{\mathrm{target}}-x_{c,N}\right)}^{2}+{\left(y_{\mathrm{target}}-y_{c,N}\right)}^{2}}$$

where (*x_c_*_,*N*_, *y_c_*_,*N*_) denotes the terminal position of the candidate trajectory after *N* prediction steps. In addition, *θ_target_* represents the relative angular error between the heading angle at the estimated candidate pose and the direction from that pose to the look-ahead target.

*θ_target_* = *tan*^−1^((*y_target_* − *y_c_*_,*N*_)/(*x_target_* − *x_c_*_,*N*_)) − *θ_c_*_,*N*_ (21)

**Figure 10:**
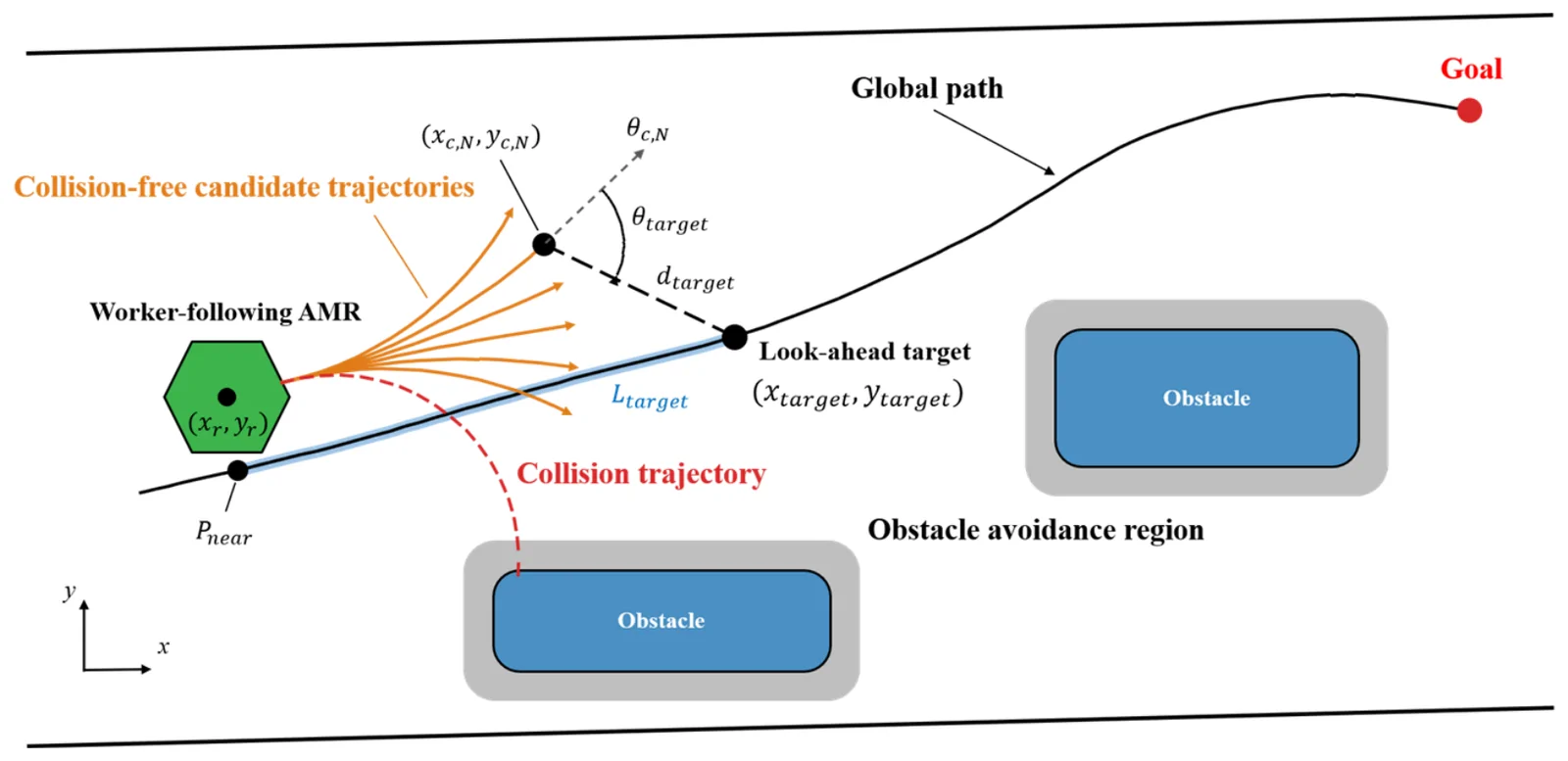
Schematic diagram of the proposed DWA (gray colored area means obstacle avoidance region).

By using both *d_target_* and *θ_target_*, the distance and heading difference between the AMR and the look-ahead target can be evaluated to determine how well the AMR is aligned with the target. This evaluation also enables the AMR to return to the global path after temporarily deviating from it to avoid obstacles, as shown in Figure 10.

The third term *C_l_* denotes the largest cost among the local costmap nodes that the candidate trajectory passes through, so that it grows as the trajectory approaches an obstacle. If the resulting *C_l_* exceeds a threshold *C_th_*, the corresponding candidate is eliminated. This process removes trajectories that pose a risk of collision with obstacles, leaving only candidate linear and angular velocities that allow the AMR to travel through safe regions. This filtering process is expressed as follows.

*V_valid_* = {(*v_c_*, *ω_c_*)| (*v_c_*, *ω_c_*) ∈ *V_r_ and C_l_*(*v_c_*, *ω_c_*) < *C_th_*} (22)

Adjusting the linear velocity and turning radius according to path curvature and look-ahead geometry can improve navigation stability in corner sections [26]. For this, the final cost function term *C_corner_* is designed to directly regulate the linear velocity by accelerating or decelerating the AMR according to the upcoming direction of the path.

Reducing the linear velocity during curved motion decreases the turning radius of the AMR, thereby reducing the risk of collision. Therefore, the cost function *C_corner_* can be expressed as follows.

(23) $$C_{\mathrm{corner}}=\left|\frac{\left|v_{c}\right|\left|\theta_{c,k+2}-\theta_{c,k+1}\right|}{L_{\mathrm{corner}}}-\left|\omega_{c}\right|\right|$$

Here, *θ_c_*_,*k*+1_ and *θ_c_*_,*k*+2_ represent the AMR heading angles at the positions reached after prediction horizons of ∆*t* and 2∆*t*. In addition, *L_corner_* represents the maximum path length that the AMR can travel over the prediction horizon of 2∆*t*. This *C_corner_* makes it possible to determine whether the AMR is approaching a curved section or a U-turn and to reduce its linear velocity accordingly.

Finally, the proposed DWA selects the optimal velocity combination with the lowest score among the valid candidate velocities as the final control command, as follows.

(24) $$\left(v^{*},\omega^{*}\right)=\arg\underset{\left(v_{c},\omega_{c}\right)\in V_{\mathrm{valid}}}{\min}G\left(v_{c},\omega_{c}\right)$$

As a result, through the evaluation metrics of the improved DWA, velocity commands can be generated by simultaneously considering global path-following performance and navigation safety even in worker-following situations.

## 6. Experiments

### 6.1. Experimental Setup

To implement the proposed algorithm, Ubuntu 22.04 is used as the Linux operating system, and ROS 2 Humble is employed as the middleware for data processing. In addition, an NVIDIA Jetson AGX Orin with 64 GB of memory is utilized as the edge computing device.

**Figure 11:**
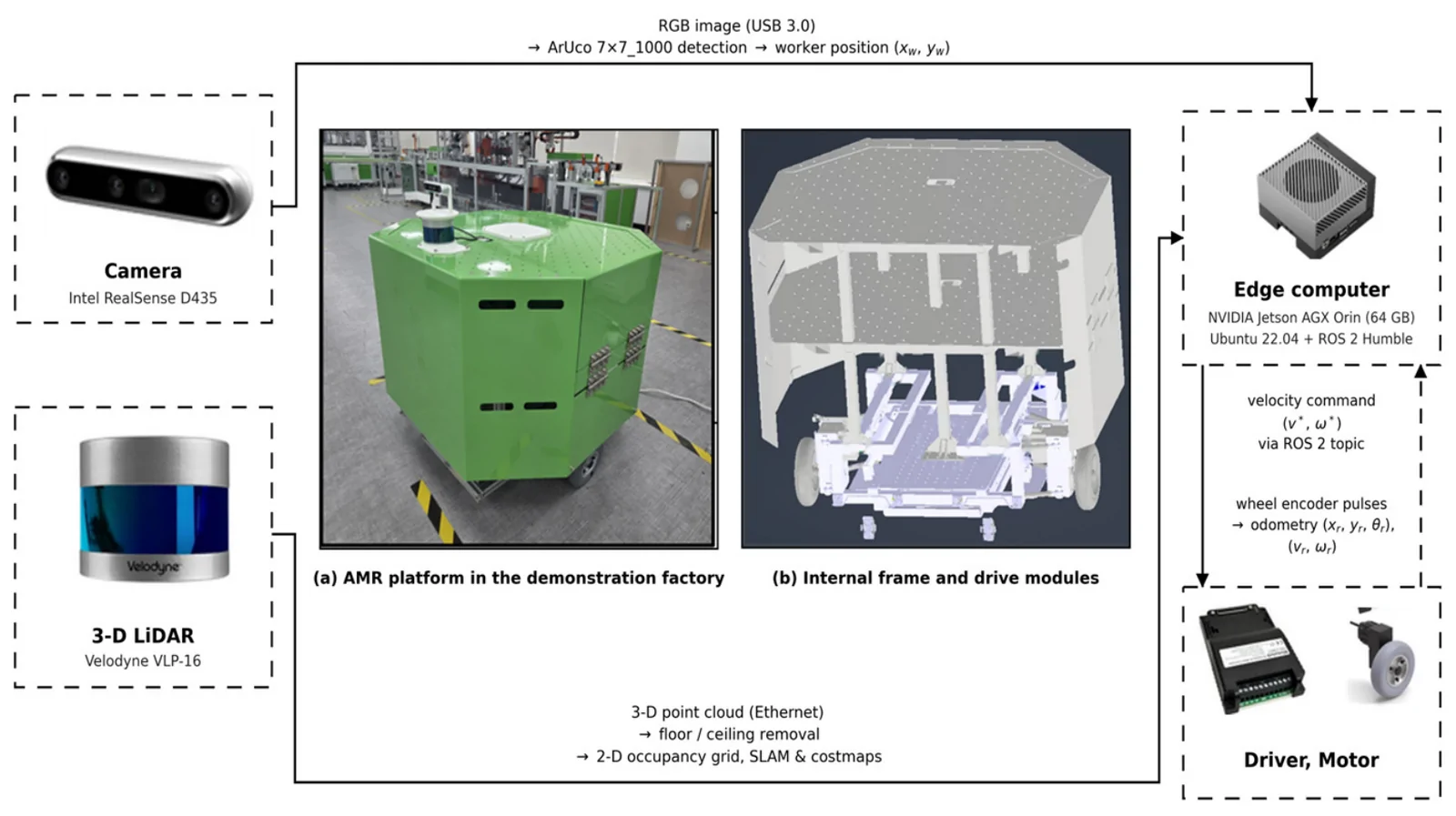
Hardware configuration of the AMR: (**a**) AMR platform in the demonstration factory; (**b**) Internal frame and drive modules.

As shown in Figure 11, a Velodyne VLP-16 3-D LiDAR (Ouster, Inc., San Francisco, CA, USA) is used to detect obstacles at various heights and thereby reduce the risk of collision, while an Intel RealSense D435 camera (RealSense, Inc., Cupertino, CA, USA) is used for ArUco marker detection. The edge computer receives the point cloud over Ethernet and the RGB image over USB 3.0, and transmits the resulting velocity command to the motor drivers, whereas the wheel encoders provide the odometry used by the DWA.

The parameters of the proposed system are listed in Table 2. Each of the four cost terms in (19) is determined by a coarse grid search in the demonstration factory. Especially, the value 0.05 m^2^ of the collinearity threshold *ε* for waypoint pruning in (7) is adopted as the largest threshold for which the deviation remains below one cell. Also, the number of collisions decreases monotonically as the weight *w*_4_ increases and reaches zero at 1.20. The value 1.20 is therefore adopted as the smallest weight *w*_4_ that eliminates collisions.

The proposed algorithm is compared with two other algorithms in terms of the number of collisions, the elapsed time, the average linear velocity and the average angular velocity. Two experimental scenarios are conducted in a demonstration factory environment that closely resembles an actual industrial workplace. The demonstration factory provides diverse navigation conditions, including straight-line motion in open spaces, turning around pillars, and obstacle avoidance in areas containing multiple pieces of equipment.

#### 6.1.1. Demonstration Factory and Experimental Scenarios

The experiments were carried out in a demonstration factory of approximately 30 m × 20 m whose layout is shown in Figure 6. The layout reproduces an actual industrial workplace: an open central aisle used for transporting materials, structural pillars distributed along the aisle, and side areas occupied by machining equipment, workbenches and material pallets.

Scenario 1 evaluates navigation in the open part of the factory. Scenario 2 additionally evaluates navigation in a cluttered area. The worker follows a route of comparable length, but the route passes through a narrow passage formed by equipment on both sides, and three additional obstacles are placed close to the corners of the route, so that the AMR has to avoid obstacles while it is turning. The main characteristics of the two scenarios are compared in Table 3.

#### 6.1.2. Comparison Algorithms

In this paper, Nav2, which is provided as a ROS 2 navigation framework, and the FMM-DWA method are selected as the two comparison algorithms. To ensure a fair comparison, all three algorithms are executed on the same AMR platform, use the same occupancy grid map generated by SLAM algorithm, and share the identical robot footprint, velocity limits, acceleration limits, control period and obstacle inflation distance. Only the global path planning module and the velocity generation module differ between the three cases.

Nav2 is the standard navigation framework of ROS 2 [27]. In the configuration used here, the global path is produced by the NavFn planner, which performs a Dijkstra search on the global costmap, and the velocity commands are produced by the DWB controller, which samples trajectories in the velocity space and scores them with a set of configurable critic plugins.

Also, FMM-DWA combines the fast marching method with the DWA [12]. The fast marching method solves the eikonal equation over the grid map and produces a distance field from the goal, from which a reference direction is assigned to every node. The DWA then selects the velocity pair that minimizes the error between the current heading of the AMR and the direction assigned to the node that the AMR occupies, so that the AMR follows the direction field instead of an explicitly generated polyline.

Throughout Section 5.2, a collision is defined as any physical contact between the body of the AMR and a structure, an obstacle or a wall. Such a contact is detected by the contact bumpers of the platform and is confirmed afterwards from the recorded video. A trial in which one or more contacts occur is counted once, so that the collision counts reported below correspond to the number of trials containing at least one contact out of the 30 trials of each scenario.

### 6.2. Experimental Results

#### 6.2.1. Statistical Analysis

**Figure 12:**
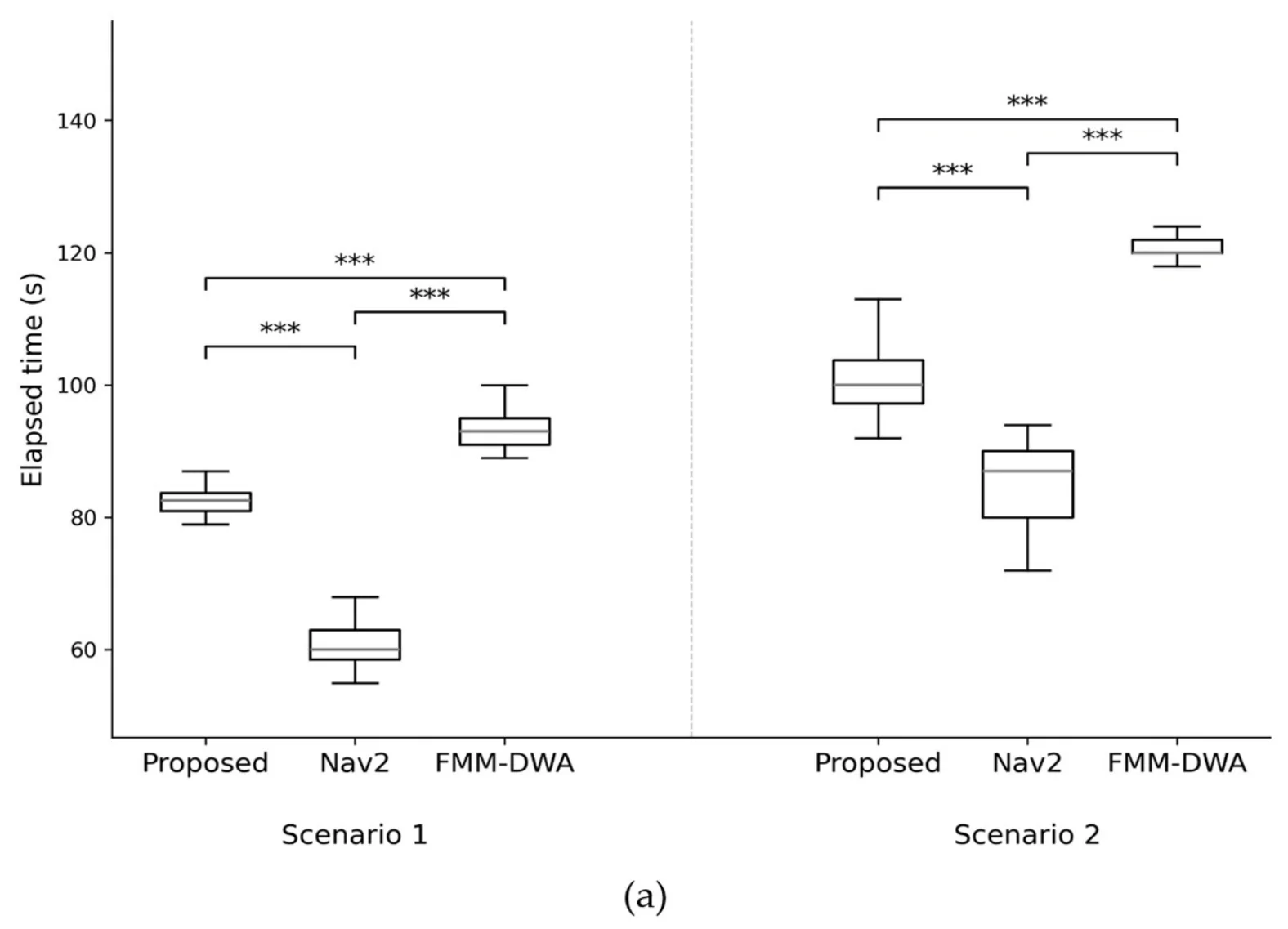

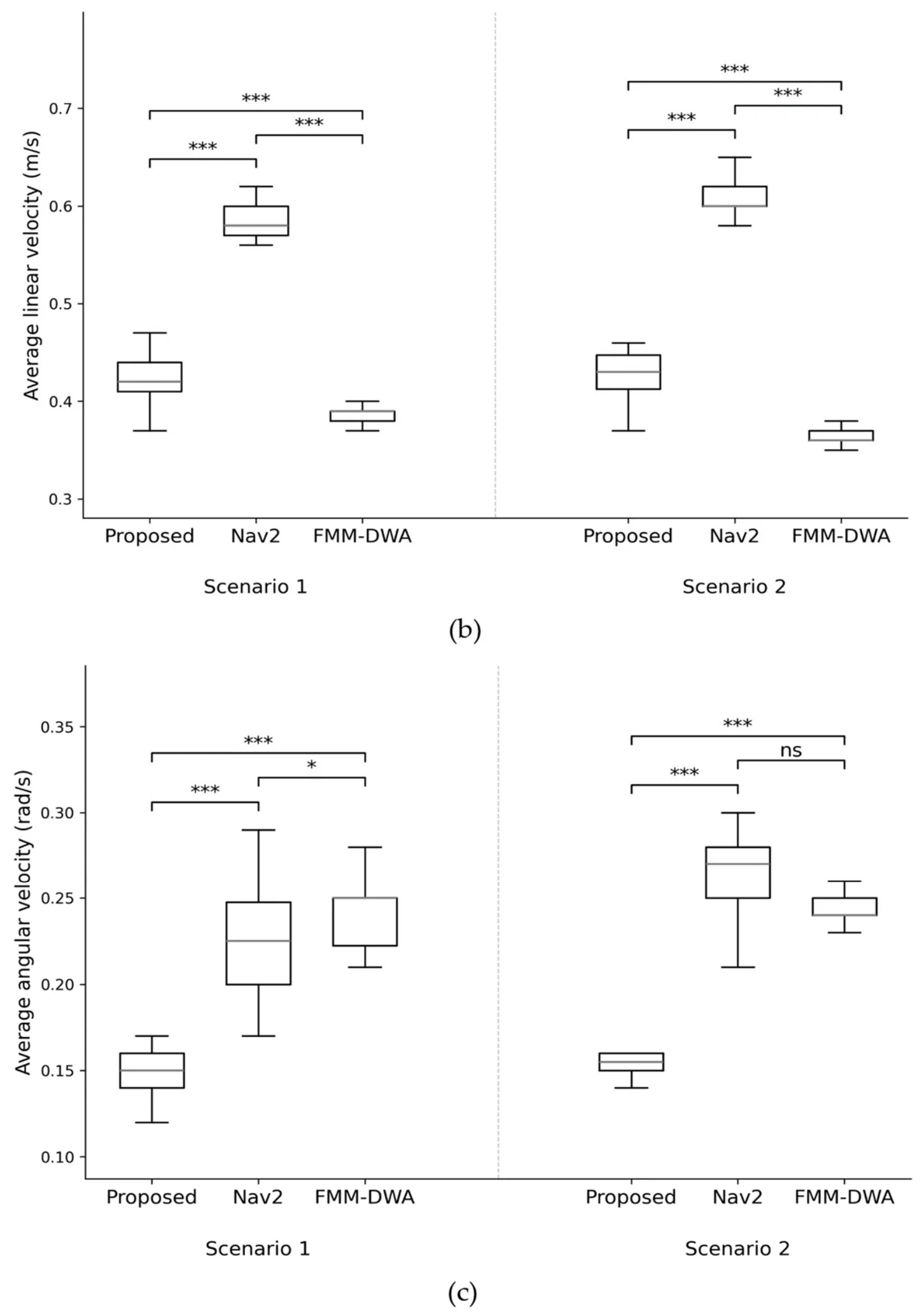
Statistical comparison of the evaluation metrics over 30 trials in each scenario. Statistical significance is denoted by *p* < 0.05 (*), *p* < 0.01 (**), *p* < 0.001 (***), *p* ≥ 0.05 (ns): (**a**) Elapsed time; (**b**) Average linear velocity; (**c**) Average angular velocity.

For each of the two scenarios, the evaluation metrics are analyzed based on 30 experimental trials, and the resulting distributions are summarized in Figure 12. In terms of elapsed time, shown in Figure 12a, Nav2 is the fastest algorithm, with medians of approximately 61 s in scenario 1 and 85 s in scenario 2, whereas FMM-DWA is the slowest, with approximately 93 s and 121 s. The proposed algorithm lies between them, at approximately 82 s and 100 s. This ordering follows directly from the average linear velocity shown in Figure 12b, where Nav2 maintains about 0.58–0.61 m/s, the proposed algorithm about 0.42–0.43 m/s and FMM-DWA about 0.36–0.38 m/s. In contrast, as shown in Figure 12c, the proposed algorithm exhibits the lowest average angular velocity in both scenarios, approximately 0.15 rad/s against 0.22–0.27 rad/s for the two comparison algorithms, indicating smaller average rotational motion during navigation. Because the Shapiro–Wilk test rejects normality for several of the distributions, the two-sided Mann–Whitney *U* test is used for all pairwise comparisons, and the resulting *p*-values are adjusted for multiple comparisons by the Holm–Bonferroni correction. The significance level is set to 0.05. All comparisons between the proposed algorithm and each of the comparison algorithms are statistically significant for the three metrics, with *p* < 0.001. Between the two comparison algorithms, however, the difference in average angular velocity is only marginally significant in scenario 1 (*p* < 0.05) and not significant in scenario 2, which indicates that Nav2 and FMM-DWA produce comparable heading variations despite their clearly different velocity profiles.

**Figure 13:**
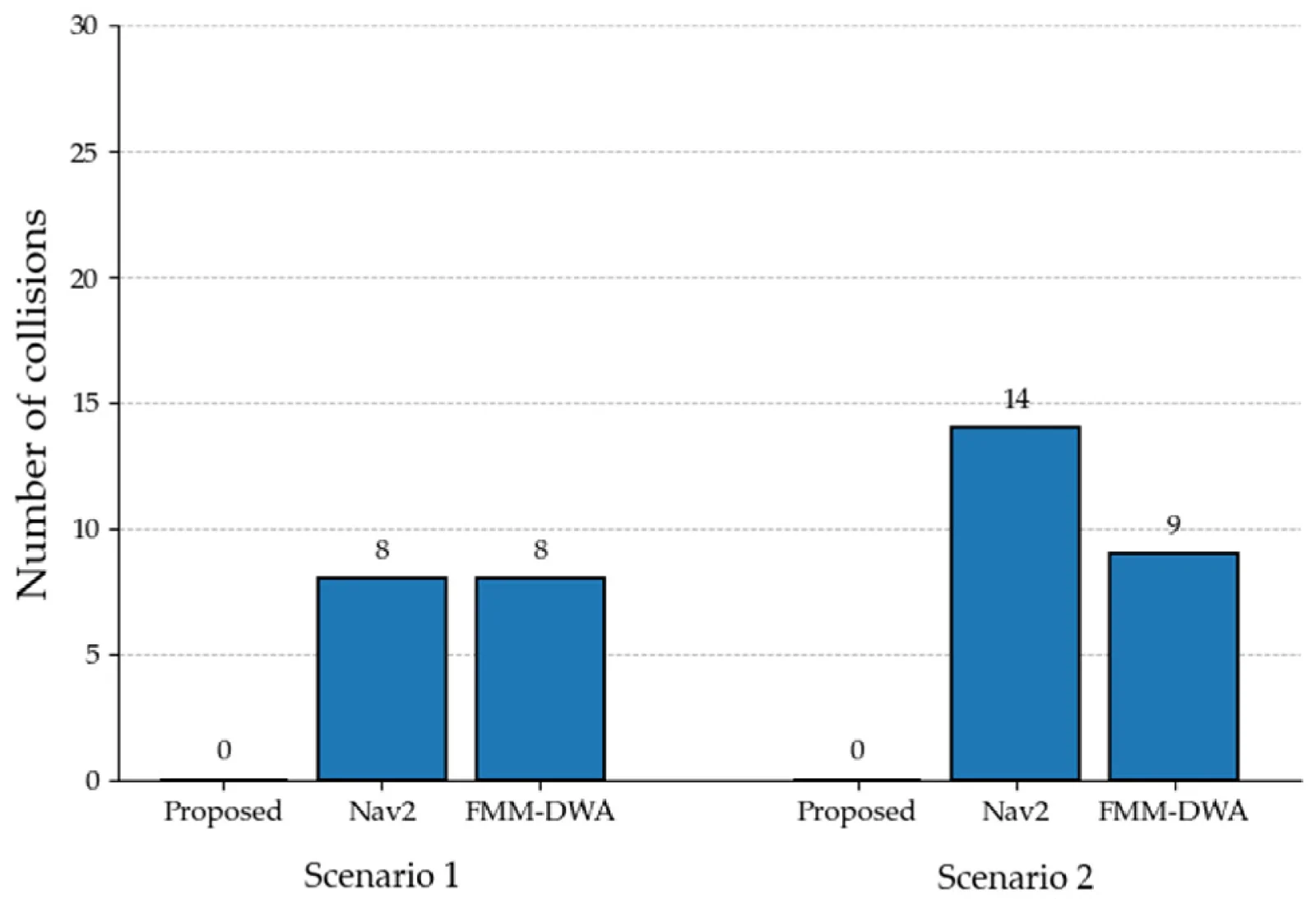
Comparison of the number of collisions in each scenario.

The collision results for each scenario are presented in Figure 13. The proposed algorithm records no collision in either scenario. In scenario 1, Nav2 and FMM-DWA each exhibit eight collisions over the 30 trials. In the more complex environment of scenario 2, the number of collisions increases for both comparison algorithms, to 14 for Nav2 and 9 for FMM-DWA, whereas the proposed algorithm again completes all 30 trials without a collision. Over the 60 trials of the two scenarios, therefore, Nav2 and FMM-DWA record 22 and 17 collisions in total, while the proposed algorithm records none.

These results indicate that although a high linear velocity can reduce the elapsed time, it may increase the number of collisions in complex industrial environments. In addition, maintaining a low linear velocity alone does not necessarily ensure safe navigation. When large variations in angular velocity occur, navigation stability may decrease, which can also increase the risk of collision.

In the case of Nav2, collisions occur most frequently in turning sections. Because the AMR maintains a relatively high linear velocity while turning, a larger turning radius is formed, increasing the probability of collision with nearby obstacles. FMM-DWA successfully passes the corner sections but shows reduced navigation stability in narrow spaces, resulting in a relatively high number of collisions. In contrast, the proposed algorithm exhibits the lowest average angular velocity and adjusts its linear velocity before entering corner sections, which allows it to pass both the corner sections and the narrow spaces without collision.

#### 6.2.2. Navigation Path Analysis for Scenario 1

**Figure 14:**
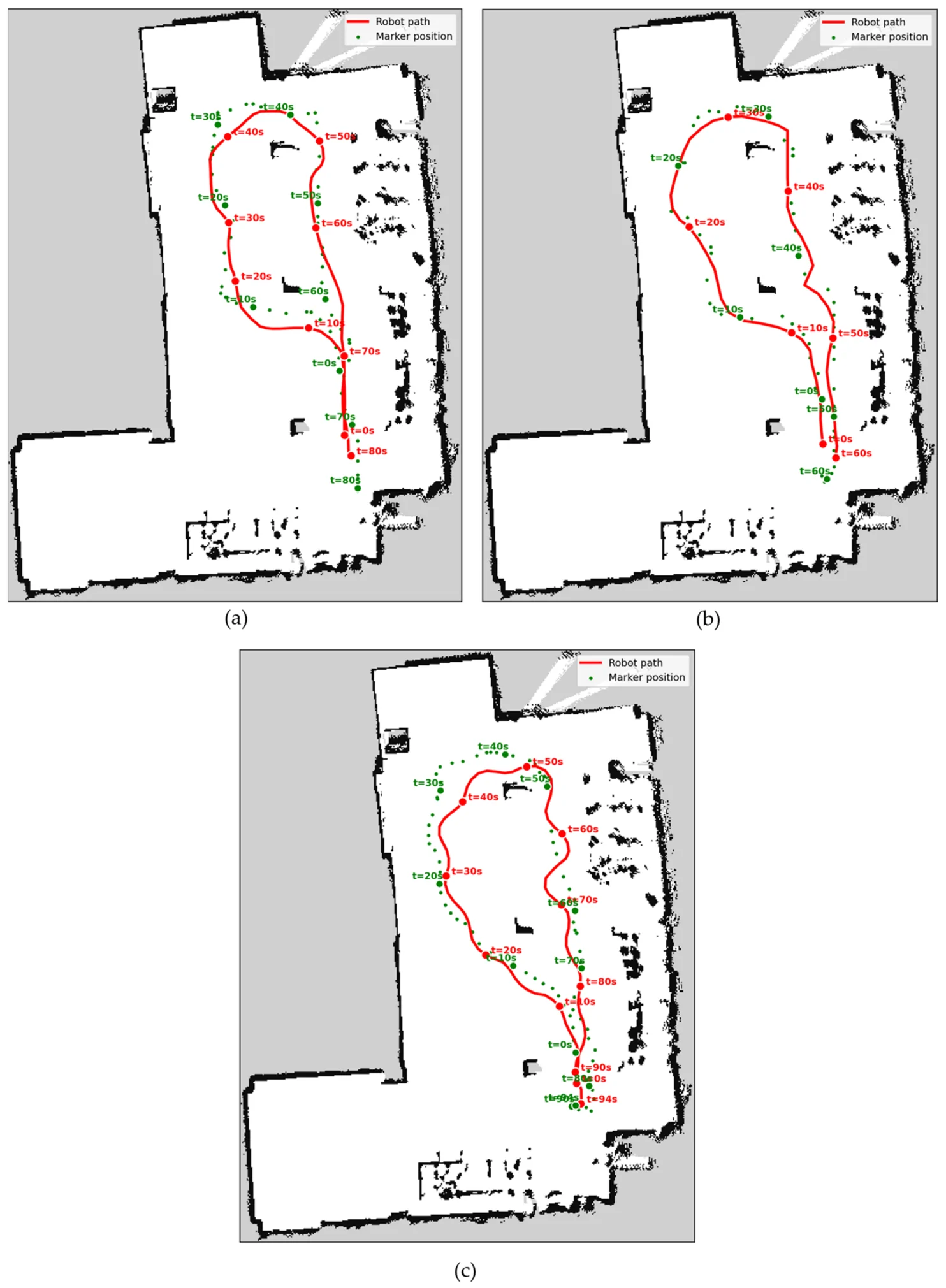
Navigation paths of one representative trial in scenario 1 for each algorithm: (**a**) Proposed; (**b**) Nav2; (**c**) FMM-DWA.

As shown in Figure 14, the results are obtained by applying the proposed algorithm, Nav2, and FMM-DWA in scenario 1. Scenario 1 represents navigation through the basic structural layout of the demonstration factory. Although the AMR generally moves through relatively open areas, some sections require it to negotiate sharp turns and pass through narrow spaces. Figure 14a–c show one representative trial for each algorithm, so the elapsed times indicated in these figures may differ slightly from the medians reported in Figure 12. In the trials shown, all three algorithms successfully complete the navigation task while tracking the worker’s position in real time.

**Figure 15:**
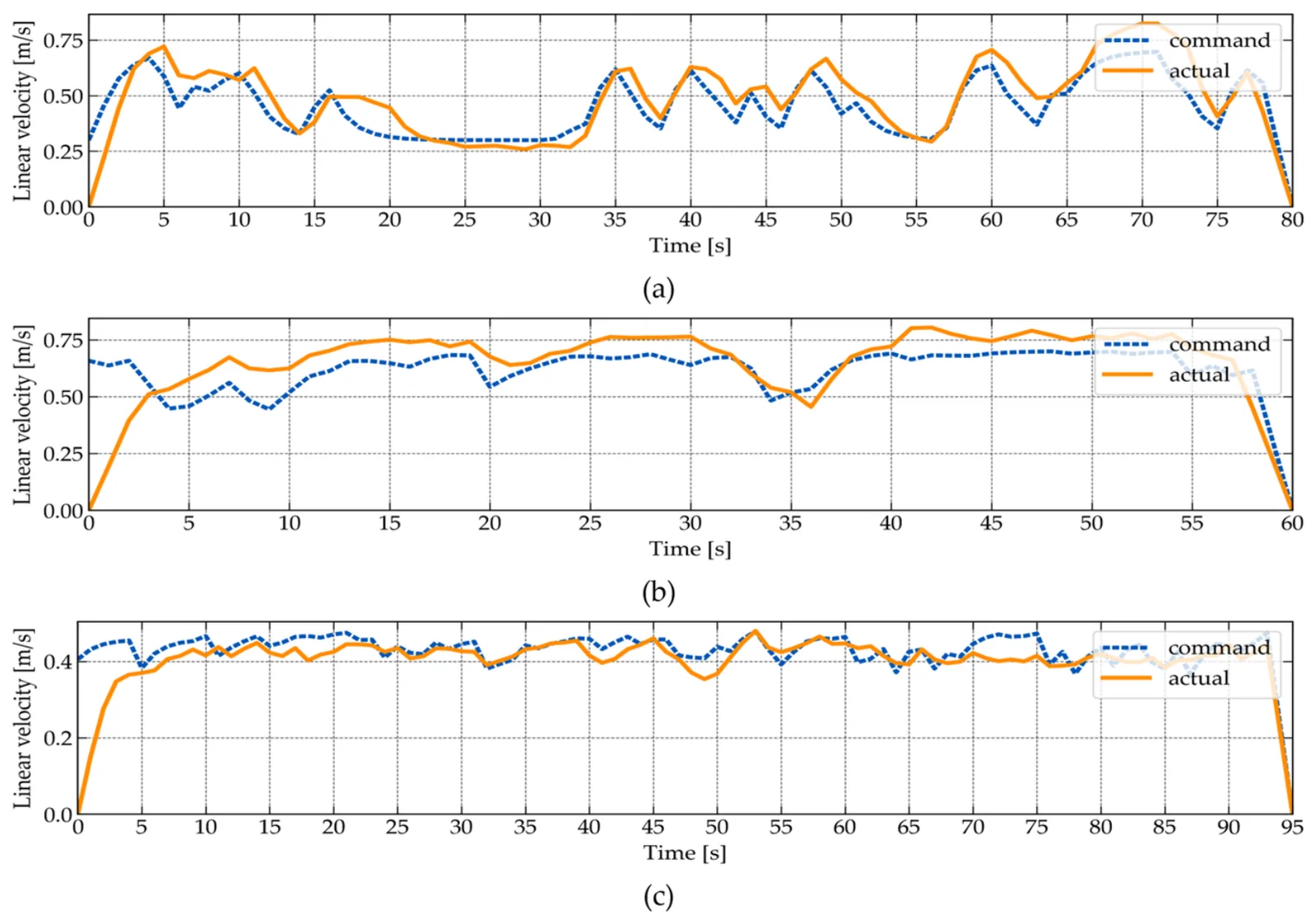
Linear velocity profiles of one representative trial in scenario 1 for each algorithm: (**a**) Proposed; (**b**) Nav2; (**c**) FMM-DWA.

As shown in Figure 15, the linear velocity profile of the proposed algorithm shows that the AMR reduces its velocity when entering curved sections or when the worker’s position changes abruptly. These results indicate that the proposed algorithm adjusts the linear velocity in response to changes in the navigation path during worker-following operation.

Nav2 generally maintains a high linear velocity throughout the navigation process. However, its trajectory contains several sections in which the AMR changes direction abruptly rather than following a smooth turn. Under such conditions, maintaining a high linear velocity may delay the initiation of turning or obstacle-avoidance maneuvers, thereby increasing the risk of collision. In fact, Nav2 records eight collisions over 30 trials.

In contrast, FMM-DWA exhibits a relatively low linear velocity and the longest navigation time. Nevertheless, it also records eight collisions. This result can be attributed to the frequent heading changes caused by its piecewise-linear trajectory characteristics. Its navigation trajectory also shows that the AMR’s heading changes repeatedly even in sections where straight-line motion is possible.

#### 6.2.3. Navigation Path Analysis for Scenario 2

**Figure 16:**
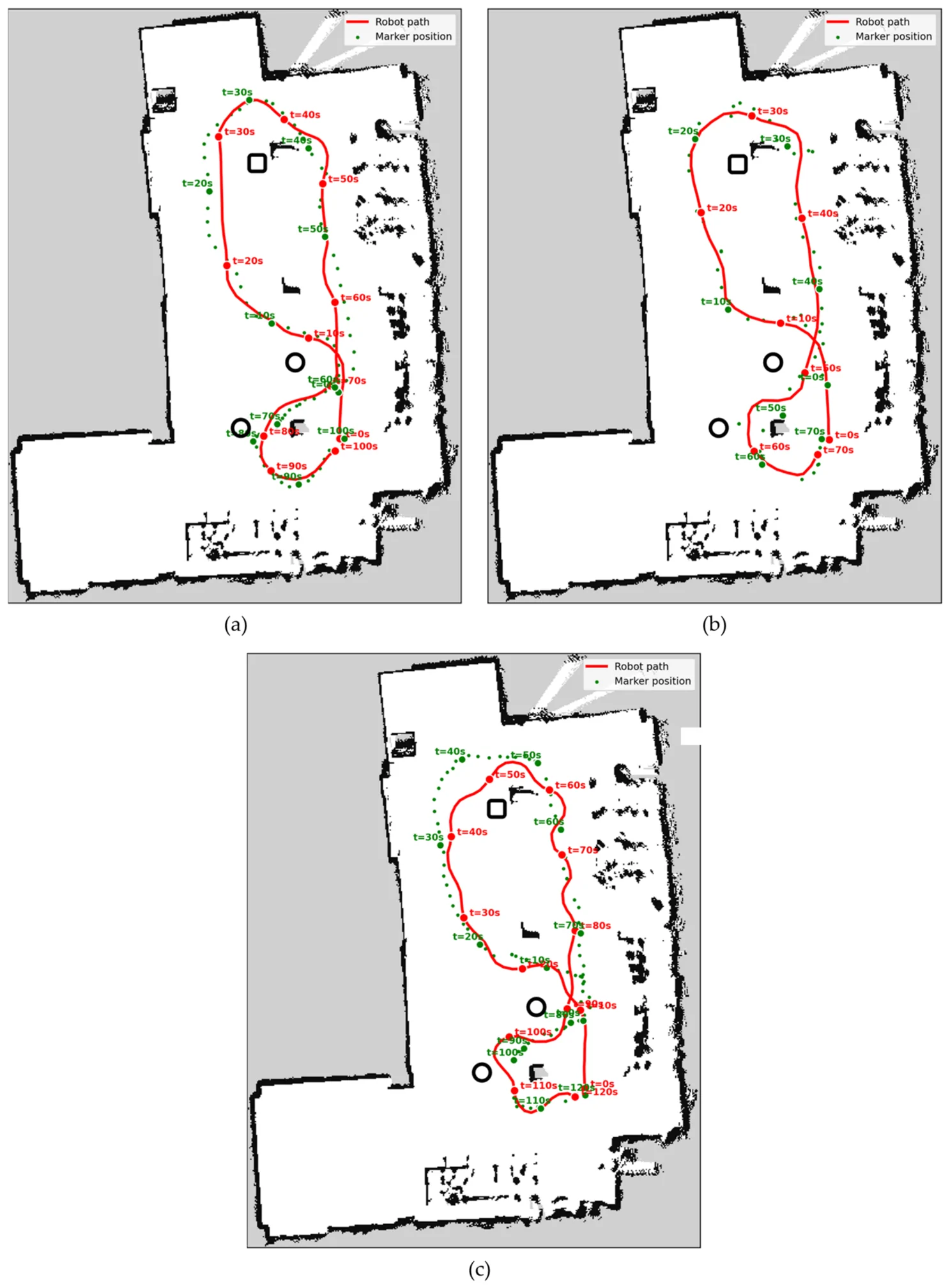
Navigation paths of one representative trial in scenario 2 for each algorithm: (**a**) Proposed; (**b**) Nav2; (**c**) FMM-DWA.

Figure 16 presents the successful navigation results for scenario 2 using each algorithm. Unlike scenario 1, three additional obstacles are placed near the navigation path in scenario 2 to evaluate navigation performance in a narrow and complex environment.

The proposed algorithm follows smooth curved trajectories in turning sections, similar to the results observed in scenario 1. In particular, in the narrow lower section of the map, which the proposed algorithm traverses between t = 70 s and t = 90 s and where most of the collisions of the comparison algorithms occur, the proposed method continuously updates the worker’s position and maintains stable tracking performance. In contrast, Nav2 passes through the same region within a relatively short time, between t = 50 s and t = 60 s, but its trajectory shows abrupt and irregular changes, and it records 14 collisions over the 30 trials. FMM-DWA successfully turns within the narrow space between t = 90 s and t = 110 s because of its low speed, and records 9 collisions. However, it exhibits excessive rotational motion relative to the global path, resulting in an increased total path length.

**Figure 17:**
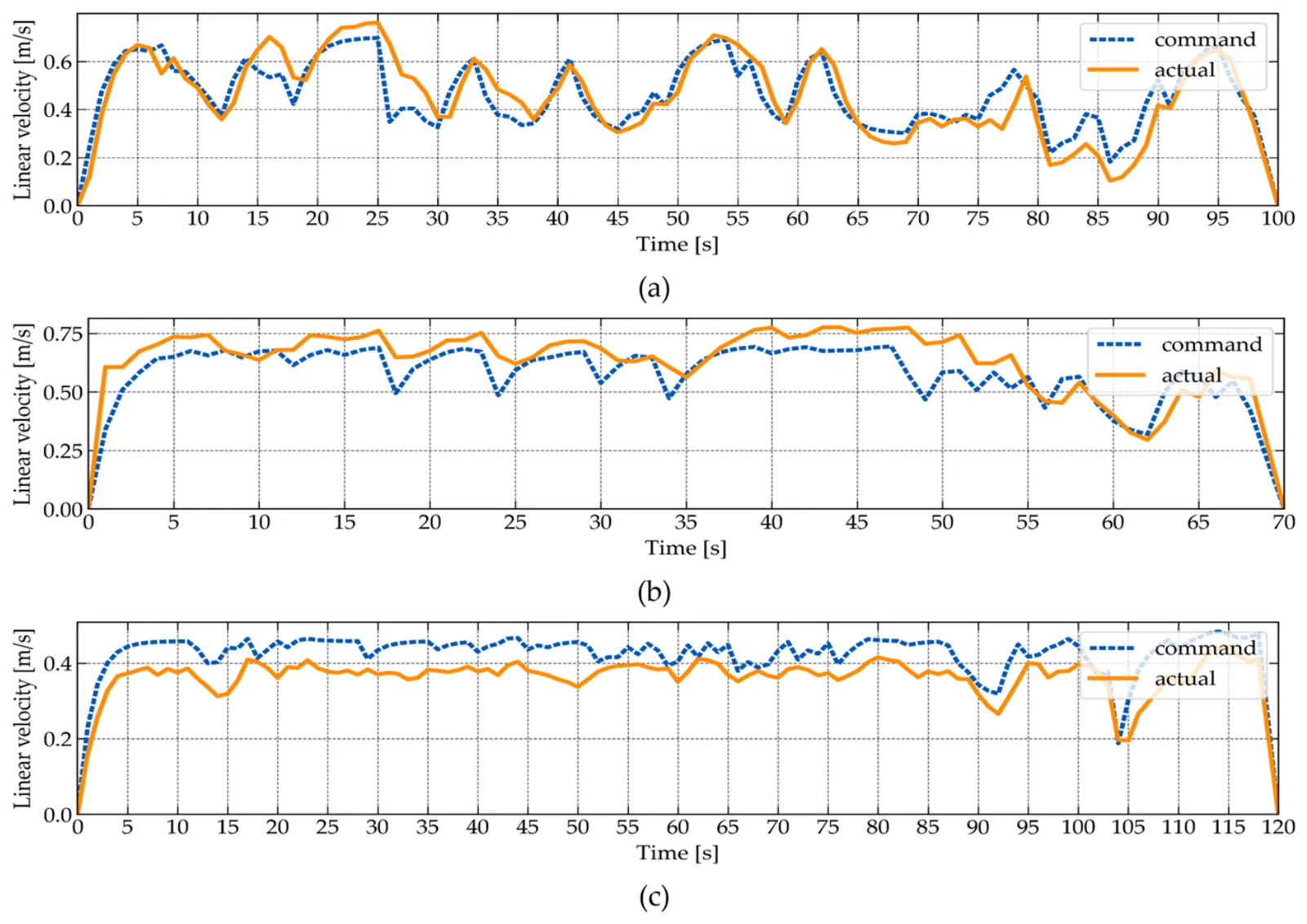
Linear velocity profiles of one representative trial in scenario 2 for each algorithm: (**a**) Proposed; (**b**) Nav2; (**c**) FMM-DWA.

This behavior can also be observed in the linear velocity profiles, as shown in Figure 17. The proposed algorithm starts to reduce its linear velocity between t = 65 s and t = 70 s, before entering the corner, and then decelerates further, to below 0.2 m/s at approximately t = 85 s, while negotiating the narrow section. In contrast, Nav2 maintains approximately 0.7 m/s and begins to reduce its actual linear velocity only after entering the corner, at approximately t = 52 s. FMM-DWA does not exhibit such a distinct deceleration phase, because the direction field keeps its commanded velocity close to 0.45 m/s over the whole run; its actual velocity therefore stays around 0.38 m/s and drops only locally, near t = 90 s and t = 105 s. As a result, only the proposed algorithm maintains a relatively high linear velocity in straight or open sections while reducing its speed before entering narrow or complex areas.

## 7. Conclusions

This paper presented a worker-following AMR navigation system that combines ArUco marker-based worker recognition, costmap-based A* global path planning refined by waypoint pruning and cubic B-spline smoothing, and an improved DWA whose cost function additionally regulates the linear velocity according to the geometry of the path ahead. The system was implemented on an AMR equipped with a 3-D LiDAR and a camera and was validated in a demonstration factory through two scenarios of 30 trials each. In these experiments, the proposed algorithm completed all 60 trials without a collision, whereas Nav2 and FMM-DWA recorded 22 and 17 collisions, respectively. It also achieved the lowest average angular velocity, approximately 0.15 rad/s against 0.22–0.27 rad/s for the comparison algorithms, while its elapsed time remained between that of the fastest algorithm, Nav2, and that of the slowest, FMM-DWA.

These results demonstrate, under the tested conditions, that the AMR can follow a worker without collision even in environments where the worker’s position continuously changes and various surrounding structures are present. Since the feasibility of the proposed method is verified using an actual AMR platform, it can serve as fundamental technology for future industrial environments in which workers and AMRs collaborate.

Future work will focus on improving the worker recognition method so that the worker can be continuously tracked even when the ArUco marker is occluded or moves outside the camera’s field of view. In addition, expanding the experiments to various industrial layouts is expected to improve the field applicability and navigation reliability of the proposed system.

## Figures and Tables

**Table 1:** Comparison of representative AMR navigation methods.

Ref.	Category	Global Planning	Local Planning	Validation
[5]	Global and Local Fusion	Improved A*	DWA with path yaw term	Simulation
[6]		Smoothed A*	Improved DWA	Forklift AGV
[7]		Reference path	Path-following control	Field vehicle
[8]	Deep learning	Mapless	Deep reinforcement learning	Simulation
[9]			Learned policy	Simulation
[11]	Direction field	Guiding vector field	Heading alignment	Small robot
[12]		FMM field	FMM-DWA	Indoor robot

**Table 2:** Parameters of the proposed worker-following AMR system.

Symbol	Description	Value
*d_max_*	Obstacle inflation distance of the global and local costmaps	0.60 m
*ε*	Collinearity threshold for waypoint pruning in (7)	0.05 m^2^
*v_min_,v_max_*	Linear velocity limits	0.00, 0.70 m/s
*ω_min_,ω_max_*	Angular velocity limits	−1.00, 1.00 rad/s
*a_v_*	Maximum linear acceleration	0.50 m/s^2^
*a_ω_*	Maximum angular acceleration	1.50 rad/s^2^
∆*t*	Control and prediction interval	0.10 s
*N*	Number of prediction steps	20
*L_target_*	Look-ahead distance in (18)	0.80 m
*L_corner_*	Maximum path length over the horizon 2Δ*t* in (23)	0.14 m
*w*_1_	Weight of the look-ahead distance term	1.00
*w*_2_	Weight of the heading error term	0.80
*w*_3_	Weight of the local costmap term	0.50
*w*_4_	Weight of the corner-aware velocity regulation term	1.20

**Table 3:** Comparison of the two experimental scenarios.

Item	Scenario 1	Scenario 2
Approximate route length of the worker	36 m	44 m
Number of turns on the route	2	4
Structural pillars adjacent to the route	2	2
Additional obstacles placed on the route	0	3
Minimum passage width	1.8 m	1.2 m
Main navigation condition	Straight following and turning around pillars	Turning combined with obstacle avoidance in a narrow passage

## Data Availability

The data presented in this study are available on request from the corresponding author.
